# Neuronal Cholesterol Deficiency Mediated by Astrocytic SREBP2 Downregulation Leads to Postoperative Cognitive Dysfunction Through Impairment of Hippocampal Synaptic Plasticity and Excitatory Synaptic Transmission

**DOI:** 10.1002/advs.202519874

**Published:** 2026-02-12

**Authors:** He Huang, Chenrui Zhou, Chen Chen, Huimei Tang, Wei Dong, Jie Wan, Weiye Kang, Ao Sun, Yiqi Liu, Chunhui Jin, Xiaobin Lyu, Yankun Zhu, Chenghua Zhou, Yuqing Wu

**Affiliations:** ^1^ Jiangsu Province Key Laboratory of Anesthesiology NMPA Key Laboratory For Research and Evaluation of Narcotic and Psychotropic Drugs Xuzhou Medical University Xuzhou China; ^2^ Department of Anesthesiology and Perioperative Medicine The First Affiliated Hospital of Nanjing Medical University Nanjing China; ^3^ Jiangsu Key Laboratory of New Drug Research and Clinical Pharmacy Xuzhou Medical University Xuzhou China

**Keywords:** excitatory synaptic transmission, neuroinflammation, postoperative cognitive dysfunction, reactive astrocyte, synaptic plasticity, SREBP2

## Abstract

Postoperative cognitive dysfunction (POCD) negatively impacts prognosis; however, the underlying mechanisms remain unclear. We demonstrated that tibial fracture surgery led to cognitive dysfunction in 18‐month‐old mice, concomitant with a reduction in hippocampal levels of cholesterol and its key metabolite 24‐hydroxycholesterol (24‐OHC). Clinically, reduced blood 24‐OHC levels were associated with cognitive decline in elderly surgery patients. Mechanistically, downregulation of sterol regulatory element‐binding protein 2 (SREBP2) in reactive astrocytes of the hippocampal dorsal CA1 (dCA1) region was an important cause of postoperative cholesterol deficiency, which in turn impaired synaptic plasticity and excitatory synaptic transmission; furthermore, this deficit could be rescued by direct cholesterol replenishment in the dCA1. Importantly, we established multiple effective therapeutic strategies—astrocyte‐specific SREBP2 overexpression, chemogenetic suppression of reactive astrocytes, and minocycline administration—all of which effectively reversed surgery‐induced cholesterol loss, alleviated synaptic dysfunction, and ultimately improved cognitive performance. Taken together, our findings not only position astrocytic SREBP2 as a promising therapeutic target for POCD but also highlight the potential diagnostic value of monitoring brain cholesterol metabolism, though this requires validation in larger longitudinal cohorts.

## Graphical abstract

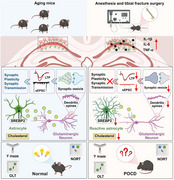

## 1 Introduction

With the rapid increase in the global aging population, the number of elderly patients undergoing surgical treatment is increasing. Postoperative cognitive dysfunction (POCD), a common neurological complication, has a high incidence rate in elderly patients undergoing surgery [1, 2]. The main clinical manifestations of POCD include impaired learning and memory, disorientation, attention deficits, and progression to irreversible cognitive impairment in severe cases. POCD also markedly increases postoperative complications, imposes a heavy economic burden on the family, and leads to unfavorable postoperative outcomes and increased mortality rates [3], making it a global public health concern.

Impaired hippocampal synaptic plasticity and neuronal excitatory inhibition are pivotal pathological mechanisms in POCD; [4, 5] however, the underlying molecular basis remains unclear. Cholesterol plays critical roles in plasma membrane fluidity, synaptic vesicle formation, and synaptic plasticity [6, 7, 8], and reductions in cholesterol level and synthesis rate are strongly associated with the aging brain and neurodegenerative diseases [9, 10]. Due to the existence of the blood‐brain barrier (BBB), brain cholesterol metabolism is separated from peripheral circulation [11, 12], and the cholesterol required by neurons in adults is predominantly synthesized by astrocytes [13, 14]. As disordered cholesterol synthesis in astrocytes in the aging brain further worsens cognitive impairment, we investigated whether cholesterol deficiency mediates the pathogenesis of POCD.

Cholesterol synthesis is tightly regulated by sterol regulatory element‐binding protein 2 (SREBP2) [15]. When cholesterol production is insufficient, sterol regulatory element‐binding transcription factor 2 (SREBF2) induces the translation of SREBP2, which is then translocated to the Golgi apparatus, along with SREBP cleavage activation protein (SCAP), and cleaved by two proteases at site 1 (S1P) and site 2 (S2P). Subsequently, the active N‐terminal fragment N‐SREBP2 moves into the nucleus and binds to sterol response elements (SREs) in the promoters of cholesterol biosynthesis genes, such as 3‐hydroxy‐3‐methylglutaryl‐CoA reductase (*HMGCR*) and 3‐hydroxy‐3‐methylglutaryl‐CoA synthase (*HMGCS*), and cholesterol uptake genes, such as low‐density lipoprotein (*LDL*) and very‐low‐density lipoprotein (*VLDL*) [16]. SREBP2 knockdown in astrocytes can inhibit cholesterol biosynthesis, impair brain development, and disrupt cognitive and motor functions [17]. This suggests that astrocytic SREBP2 is a central link in the regulation of cholesterol metabolism in the brain.

Reactive astrocytes are strongly induced by pathological stimuli such as peripheral injury, and studies show that reactive astrocyte‐mediated neuroinflammation contributes to POCD; [18, 19] however, the associated changes in the biological functions of reactive astrocytes remain unclear. The decline in neurotrophic support from reactive astrocytes in neurodegenerative diseases has received increasing attention [20]. Single‐cell transcriptome sequencing studies have demonstrated that *SREBF2* mRNA levels in reactive astrocytes are significantly downregulated in the elderly and in patients with cognitive impairment [21], convincingly suggesting a close connection between disrupted cholesterol metabolism and the pathogenesis of neurodegenerative diseases. However, it remains unclear whether anesthesia/surgery disrupts astrocytic cholesterol metabolism, particularly SREBP2‐mediated cholesterol synthesis, in the hippocampus of aged mice, and whether this disruption acts as a key pathogenic intermediary connecting astrocytic neuroinflammation to cognitive decline in POCD.

To investigate this possibility, we established a surgical model of tibial fracture (TF) in elderly mice and analyzed peripheral blood samples from elderly patients undergoing orthopedic surgery to investigate the role of brain cholesterol deficiency in POCD. To clarify the direct causal link, we employed two complementary strategies: direct manipulation of SREBP2 in astrocytes and pharmacological inhibition of neuroinflammation with minocycline. Our results support the hypothesis that neuronal cholesterol deficiency caused by SREBP2 downregulation in reactive astrocytes is an important factor leading to hippocampal synaptic impairment and cognitive dysfunction.

## 2 Results

### 2.1 Anesthesia/Surgery Caused Postoperative Cognitive Impairment in Elderly Mice

**FIGURE 1:**
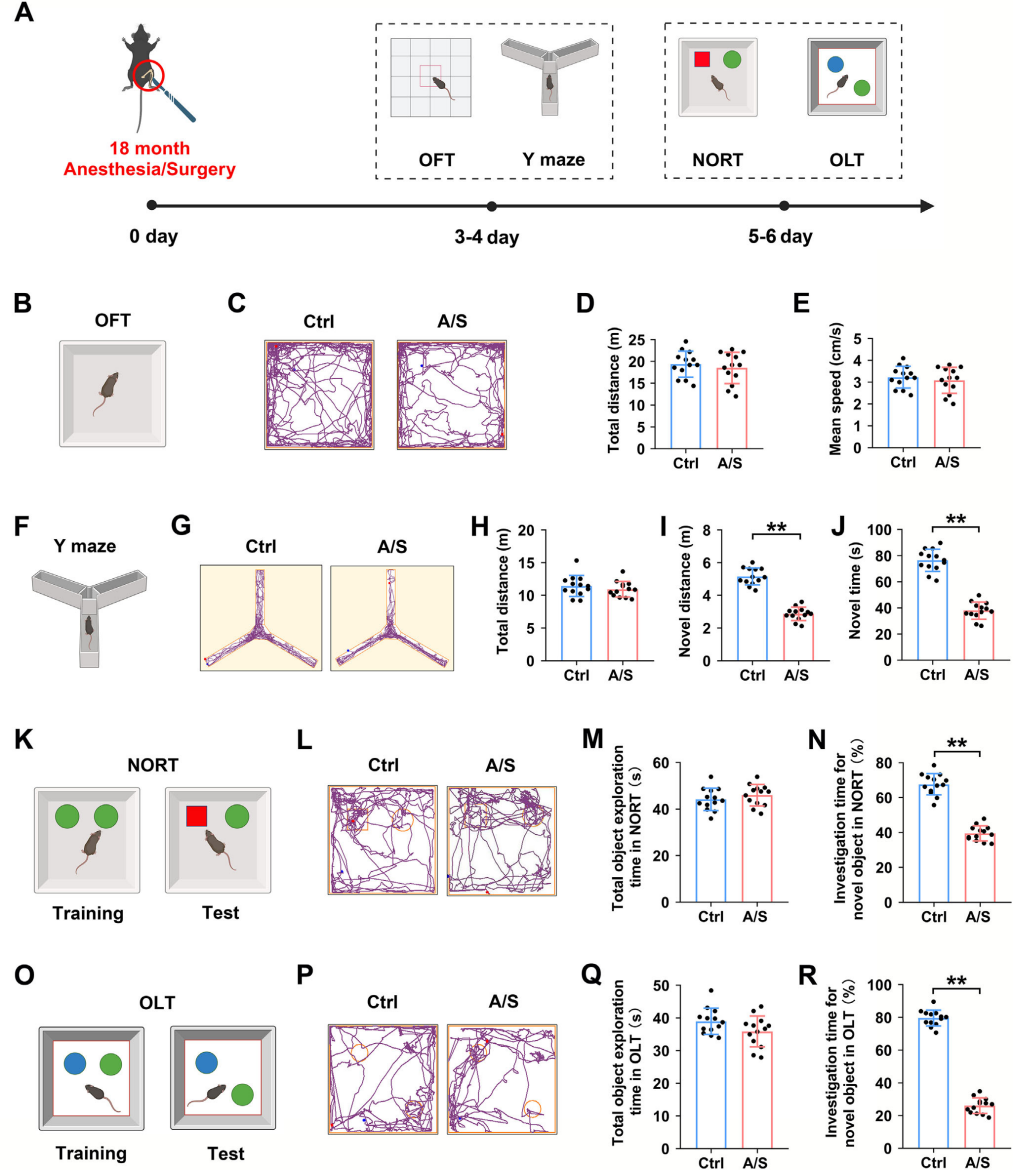
Anesthesia/surgery‐induced cognitive impairment in elderly mice. (A) Multiple behavioral tests were conducted from day 3 to day 6 after tibial fracture surgery. (B) Diagrammatical representation of the open field test (OFT). (C) Representative trajectory in the OFT. (D) Comparison of total moving distance between the Ctrl and AS groups (*n* = 13 mice/group). Equal variances were confirmed using the F‐test [F(12, 12) = 1.432, *p* = 0.5439]. The data were normally distributed; *p*‐values were calculated using the unpaired *t*‐test. (E) Comparison of average speeds between the two groups (*n* = 13 mice/group). Equal variances were confirmed using the F‐test [F(12, 12) = 1.432, *p* = 0.5439]. The data were normally distributed; *p*‐values were calculated using the unpaired *t*‐test. (F) Diagrammatical representation of the Y‐maze test. (G) Representative trajectory in the Y‐maze test. (H) Comparison of total moving distance between the two groups (*n* = 13 mice/group). Equal variances were confirmed using the F‐test [F(12, 12) = 1.798, *p* = 0.3228]. The data were normally distributed; *p*‐values were calculated using the unpaired *t*‐test. (I) Comparison of total moving distance in the novel arm between the groups (*n* = 13 mice/group). Equal variances were confirmed using the F‐test [F(12, 12) = 1.646, *p* = 0.4004]. The data were normally distributed; *p*‐values were calculated using the unpaired *t*‐test. (J) Comparison of time spent in the novel arm between the groups (*n* = 13 mice/group). Equal variances were confirmed using the F‐test [F(12, 12) = 1.691, *p* = 0.3757]. The data were normally distributed; *p*‐values were calculated using the unpaired *t*‐test. (K) Diagrammatical representation of the novel object recognition test (NORT). (L) Representative trajectory in the NORT. (M) Comparison of the total object exploration time between the two groups (*n* = 13 mice/group). Equal variances were confirmed using the F‐test [F(12, 12) = 1.099, *p* = 0.8729]. The data were normally distributed; *p*‐values were calculated using the unpaired *t*‐test. (N) Comparison of percentage of time spent exploring a novel object between the two groups (*n* = 13 mice/group). Equal variances were confirmed using the F‐test [F(12, 12) = 1.994, *p* = 0.2462]. The data were normally distributed; *p*‐values were calculated using the unpaired *t*‐test. (O) Diagrammatical representation of the object location test (OLT). (P) Representative trajectory in the OLT. (Q) Comparison of total object exploration time between the two groups (*n* = 13 mice/group). Equal variances were confirmed using the F‐test [F(12, 12) = 1.402, *p* = 0.5678]. The data were normally distributed; *p*‐values were calculated using the unpaired *t*‐test. (R) Comparison of percentage of time spent exploring a novel object between the two groups (*n* = 13 mice/group). Equal variances were confirmed using the F‐test [F(12, 12) = 1.018, *p* = 0.9759]. The data were normally distributed; *p*‐values were calculated using the unpaired *t*‐test. All data are presented as mean ± SD values; **p < 0.01 *vs* the Ctrl group.

The flowchart of the behavioral experiments is presented in Figure 1A. The OFT was performed on the third day after surgery to evaluate motor ability (Figure 1B). Figure 1C shows the representative trajectories of mice in the Ctrl and A/S groups. Total movement distance and average movement speed did not differ significantly between the two groups (*n* = 13 mice/group, *t*‐test, *t*_24_ = 0.6773, *p *= 0.5047, Figure 1D; *n* = 13 mice/group, *t*‐test, *t*_24_ = 0.6773, *p *= 0.5047, Figure 1E). This suggests that surgery did not affect the motor abilities of the mice. Following the OFT, the Y‐maze test was conducted on postoperative day 4 (Figure 1F). As shown in the representative trajectory of the experiment (Figure 1G), there was no remarkable difference in total movement distance between the two groups(*n* = 13 mice/group, *t*‐test, *t*_24 _= 0.894, *p *= 0.3802, Figure 1H); however, the movement distance and time spent in the new arm were significantly reduced in the A/S group (*n* = 13 mice/group, *t*‐test, *t*_24 _= 12.54, *p *< 0.01, Figure 1I; *n* = 13 mice/group, *t*‐test, *t*_24 _= 12.93, *p *< 0.01, Figure 1J). The NORT and OLT were conducted on the postoperative days 5–6 (Figure 1K,O). Figure 1L shows the representative trajectory in the NORT. No significant difference was observed in the total time spent exploring two objects between the two groups (*n* = 13 mice/group, *t*‐test, *t*_24 _= 0.9663, *p *= 0.3435, Figure 1M), while the percentage of investigation time for a novel object was lower in the A/S group (*n* = 13 mice/group, *t*‐test, *t*_24 _= 13.5, *p *< 0.01, Figure 1N). Figure 1P shows the representative trajectory in the OLT. There was no statistical difference in the total time spent exploring two objects between the two groups (*n* = 13 mice/group, *t*‐test, *t*_24 _= 1.809, *p *= 0.083, Figure 1Q), while the percentage of time spent exploring an object in a new location was significantly lower in the A/S group (*n* = 13 mice/group, *t*‐test, *t*_24 _= 28.33, *p *< 0.01, Figure 1R). Thus, the behavioral results indicated that anesthesia/surgery induced neurocognitive dysfunction in elderly mice.

### 2.2 Characteristics of the Hippocampal Transcriptome Dataset in POCD Mice That Underwent TF Surgery

**FIGURE 2:**
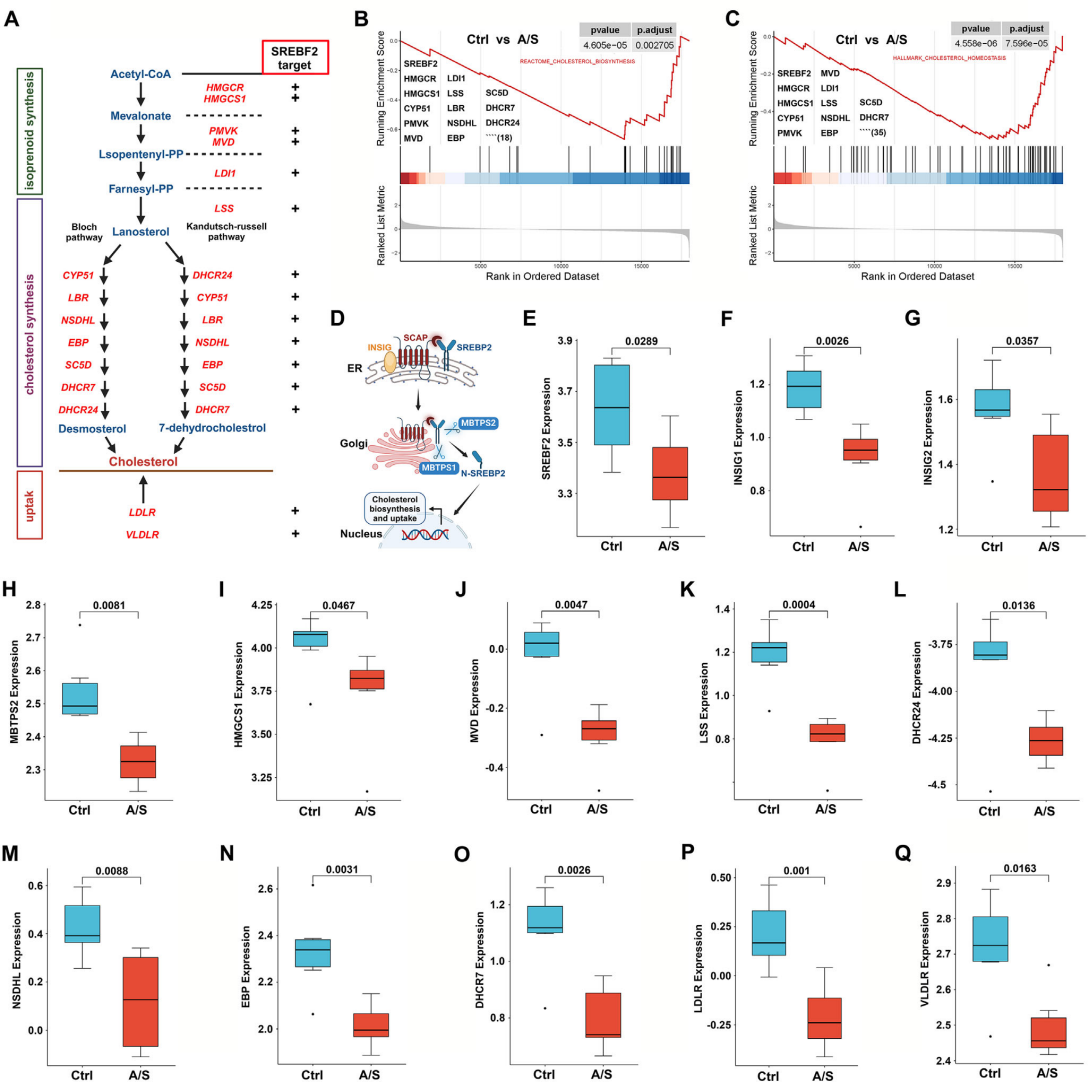
Characteristics of the hippocampal transcriptome dataset of POCD mice that underwent tibial fracture surgery. (A) Key enzymes in the cholesterol biosynthesis and uptake pathways. (B) Enrichment analysis based on REACTOME_CHOLESTEROL_BIOSYNTHESIS showed that the core genes were significantly inhibited after anesthesia/surgery. (C) Enrichment analysis based on HALLMARK_CHOLESTEROL_HOMEOSTASIS showed that the core genes were significantly inhibited after anesthesia/surgery. (D) SREBP2, a key transcription factor in cholesterol homeostasis regulation, was found to be involved in regulating cholesterol synthesis and uptake. (E) Comparison of SREBF2 mRNA expression between the two groups. (F) Comparison of INSIG1 mRNA expression between the two groups. (G) Comparison of INSIG2 mRNA expression between the two groups. (H) Comparison of MBTPS2 mRNA expression between the two groups. (I) Comparison of HMGCS1 mRNA expression between the two groups. (J) Comparison of MVD mRNA expression between the two groups. (K) Comparison of LSS mRNA expression between the two groups. (L) Comparison of DHCR24 mRNA expression between the two groups. (M) Comparison of NSDHL mRNA expression between the two groups. (N) Comparison of EBP mRNA expression between the two groups. (O) Comparison of DHCR7 mRNA expression between the two groups. (P) Comparison of LDLR mRNA expression between the two groups. (Q) Comparison of VLDLR mRNA expression between the two groups. *n* = 6 mice/group.

Cholesterol‐related biological processes involve several steps, including cholesterol biosynthesis and uptake, with multiple key enzymes participating in the associated pathways (Figure 2A). We analyzed a published hippocampal transcriptome dataset for 12–14‐month‐old POCD mice that underwent TF surgery (GSE95426). GSEA identified a significant enrichment of the gene sets REACTOME_CHOLESTEROL_BIOSYNTHESIS (Figure 2B) and HALLMARK_CHOLESTEROL_HOMEOSTASIS (Figure 2C) in the dataset. Notably, the core enrichment genes in cholesterol‐related biological processes were significantly downregulated in the A/S group compared to the Ctrl group. Schematic of SREBP2 cleavage, nuclear translocation, and regulation of cholesterol genes (Figure 2D). The mRNA levels of *SREBF2* and its target genes *INSIG1*, *INSIG2*, *MBTPS2*, *HMGCS1*, *MVD*, *LSS*, *DHCR24*, *NSDHL*, *EBP*, *DHCR7*, *LDLR*, and *VLDLR* were significantly downregulated in the A/S group compared to those in the Ctrl group (Figure 2E–Q).

### 2.3 Anesthesia/Surgery Inhibited Cholesterol Synthesis by Decreasing SREBP2 Expression in dCA1 Astrocytes

**FIGURE 3:**
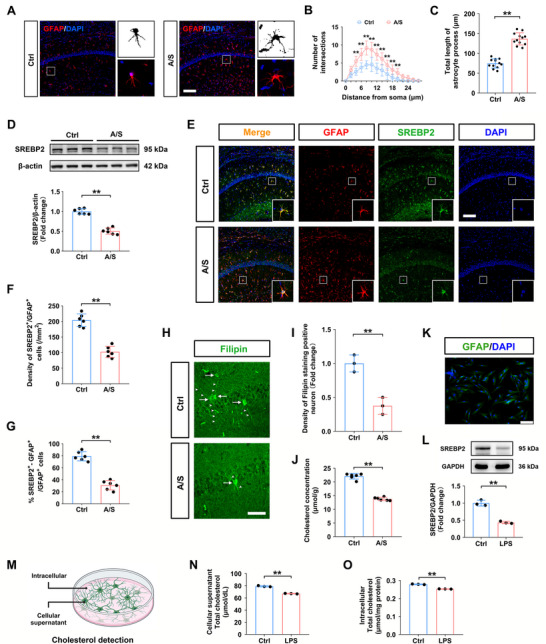
Anesthesia/surgery inhibited SREBP2 expression in astrocytes in the dCA1 of elderly mice and decreased the cholesterol levels. (A) GFAP‐positive immunofluorescence images showing dCA1 astrocytes in the Ctrl and A/S groups (20× magnification; scale bar, 100 µm). (B) Sholl analysis revealed that astrocyte processes became significantly more complex in the A/S group (*n* = 12 astrocytes from 3 mice/group). *p*‐values were calculated using two‐way ANOVA with Bonferroni's post hoc test. (C) Total astrocyte process length was higher in the A/S group (*n* = 12 astrocytes from 3 mice/group). Equal variances were confirmed using the F‐test [F(11, 11) = 1.747, *p* = 0.3687]. The data were normally distributed; *p*‐values were calculated using the unpaired t‐test. (D) Comparison of hippocampal SREBP2 level between the two groups (*n* = 6 mice/group). Equal variance was confirmed by the F‐test [F(5, 5) = 1.263, *p* = 0.8038]. The data were normally distributed; *p*‐values were calculated using the unpaired *t*‐test. (E) Representative immunofluorescence images of SREBP2 (green) and GFAP (red) in the dCA1. Cells were counterstained with DAPI (blue) (20× magnification; scale bar, 100 µm). (F) Density of SREBP2/GFAP co‐labeled cells (*n* = 6 mice/group). Equal variances were confirmed using the F‐test [F(5, 5) = 1.303, *p* = 0.7788]. The data were normally distributed; *p*‐values were calculated using the unpaired t‐test. G) Co‐labeling rate of SREBP2+/GFAP+ cells (*n* = 6 mice/group). Equal variances were confirmed using the F‐test [F(5, 5) = 1.038, *p* = 0.9684]. The data were normally distributed; *p*‐values were calculated using the unpaired *t*‐test. (H) Representative immunofluorescence images of Filipin (green) in the dCA1 (60× magnification; scale bar, 100 µm). (I) Density of Filipin positive neurons in the dCA1 (*n* = 3 mice/group). Equal variances were confirmed using the F‐test [F(2, 2) = 1]. The data were normally distributed; *p*‐values were calculated using the unpaired *t*‐test. (J) Cholesterol concentration in the hippocampus (*n* = 6 mice/group). Equal variances were confirmed using the [F(5, 5) = 2.878, *p* = 0.2708]. The data were normally distributed; *p*‐values were calculated using the unpaired *t*‐test. (K) Representative immunofluorescence images of primary hippocampal astrocytes (20× magnification; scale bar, 50 µm). (L) Comparison of SREBP2 protein expression in the primary astrocytes between Ctrl and LPS groups (*n* = 3 independent experiments/group). Equal variances were confirmed using the F‐test [F(2, 2) = 5.914, *p* = 0.2893]. The data were normally distributed; *p*‐values were calculated using the unpaired t‐test. (M) Intracellular and cellular supernatant cholesterol detection. (N) Comparison of the cellular supernatant total cholesterol between two groups (*n* = 3 independent experiments/group). Equal variances were confirmed using the F‐test [F(2, 2) = 6.143, *p* = 0.28]. The data were normally distributed; *p*‐values were calculated using the unpaired t‐test. (O) Comparison of the intracellular total cholesterol between two groups (*n* = 3 independent experiments/group). Equal variances were confirmed using the F‐test [F(2, 2) = 6.292, *p* = 0.2743]. The data were normally distributed; p‐values were calculated using the unpaired t‐test. All data are presented as mean ± SD values; **p < 0.01 vs the Ctrl group.

GFAP immunofluorescence staining showed that the morphology of dCA1 astrocytes in the A/S group became more complex (Figure 3A). Sholl analysis showed that the number of process intersections was significantly decreased in the A/S group compared with that in the Ctrl group (*n* = 12 astrocytes from 3 mice/group, two‐way ANOVA, F(1, 22) = 102.2, *p *< 0.01, Figure 3B). The total length of the astrocyte processes, which is an indirect indicator of astrocyte reactivity, in the A/S group was also longer than that in the Ctrl mice (*n* = 12 astrocytes from 3 mice/group, *t*‐test, *t*_22 _= 11.19, *p *< 0.01, Figure 3C). Western blotting revealed that SREBP2 was significantly downregulated in the A/S group compared to the control group (*n* = 6 mice/group, *t*‐test, *t*_10_ = 11.29, *p *< 0.01, Figure 3D). SREBP2/GFAP double‐immunofluorescence images of the dCA1 are shown in Figure 3E. The density of SREBP/GFAP double‐positive cells in the dCA1 and the percentage of SREBP^+^/GFAP^+^ cells were also significantly decreased in the A/S group compared with those in the Ctrl group (*n* = 6 mice/group, *t*‐test, *t*_10_ = 9.176, *p *< 0.01, Figure 3F; *n* = 6 mice/group, *t*‐test, *t*_10_ = 10.8, *p *< 0.01, Figure 3G). Neuronal cholesterol was visualized by Filipin staining, which labels neuronal bodies and axons (Figure 3H). The density of Filipin‐positive neurons in the dCA1 was significantly lower in the A/S group than in the Ctrl group (*n* = 3 mice/group, *t*‐test, *t*_4 _= 6.124, *p *< 0.01, Figure 3I). We also found that the cholesterol content in hippocampal tissue decreased significantly after surgery (*n* = 6 mice/group, *t*‐test, *t*_10 _= 17.02, *p *< 0.01, Figure 3J). These results indicated that anesthesia/surgery inhibited the expression of SREBP2 in hippocampal reactive astrocytes, resulting in postoperative cholesterol deficiency.

LPS‐induced activation of astrocytes is a commonly used cell model for studying the mechanism of POCD in vitro. Primary astrocytes were confirmed using GFAP immunofluorescence staining (Figure 3K), and SREBP2 levels were significantly decreased in LPS‐induced hippocampal astrocytes (*n* = 3 independent experiments/group, *t*‐test, *t*_4 _= 10.64, *p *< 0.01, Figure 3L). Furthermore, LPS treatment significantly reduced the cholesterol content in both the culture medium and in astrocytes (Figure 3M; *n* = 3 independent experiments/group, *t*‐test, *t*_4 _= 18.88, *p *< 0.01, Figure 3N; *n* = 3 independent experiments/group, *t*‐test, *t*_4_ = 23.78, *p *< 0.01, Figure 3O).

### 2.4 Plasma 24‐OHC Levels Were Decreased in PND Patients and Mice That Underwent Anesthesia/Surgery

**FIGURE 4:**
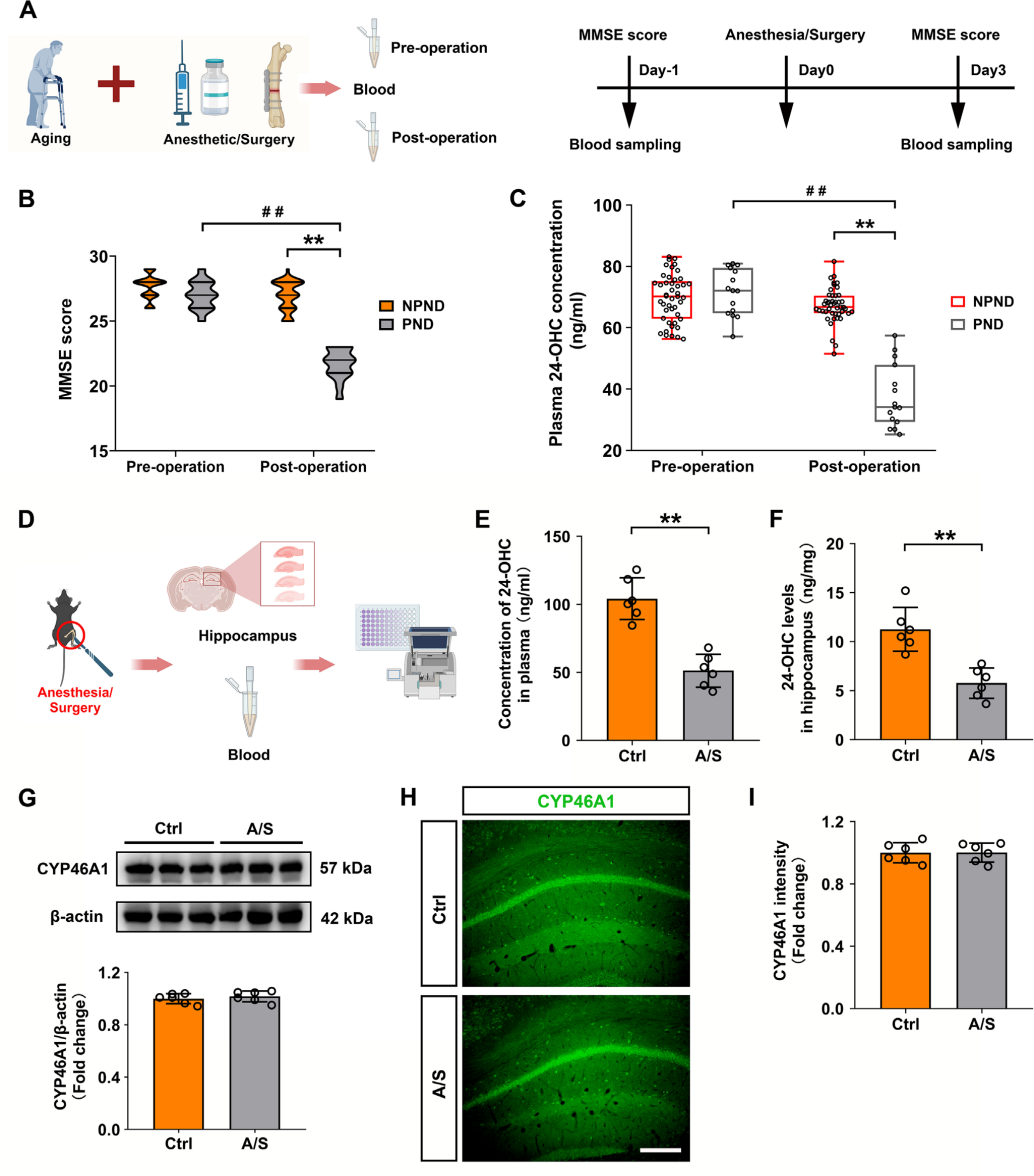
PND patients and elderly POCD mice had lower 24‐OHC levels. (A) Flowchart of the clinical assessments. B) Postoperative MMSE scores of PND patients were significantly lower than those of NPND patients. **p < 0.01. The data were not normally distributed; *p*‐values were calculated using the Mann–Whitney U test. Furthermore, PND patients showed a significant decline in postoperative MMSE scores relative to the preoperative baseline scores. ^##^ *p *< 0.01. Equal variances were confirmed using the F‐test [F(14, 14) = 1.208, *p* = 0.7282]. The data were normally distributed; *p*‐values were calculated using the unpaired *t*‐test. *n* = 15 patients in the PND group; *n* = 45 patients in the NPND group. (C) Postoperative plasma 24‐OHC concentration was significantly lower in PND patients than in NPND patients, **p < 0.01. The data were not normally distributed; *p*‐values were calculated using the Mann–Whitney U test. Moreover, the postoperative 24‐OHC concentration of PND patients was significantly lower compared with their preoperative levels. Equal variances were confirmed using the F‐test [F(14, 14) = 1.806, *p* = 0.2808]. The data were normally distributed; *p*‐values were calculated using the unpaired *t*‐test. ^##^ *p* < 0.01. *n* = 15 patients in the PND group; n = 45 patients in the NPND group. (D) Anesthesia/surgery model establishment and sample collection. (E) The plasma 24‐OHC concentration in mice decreased after anesthesia/surgery (*n* = 6 mice/group). Equal variances were confirmed using the F‐test [F(5, 5) = 1.594, *p* = 0.6213]. The data were normally distributed; *p*‐values were calculated using the unpaired *t*‐test. **p < 0.01. F) The 24‐OHC levels in the hippocampal tissue of mice decreased after anesthesia/surgery (*n* = 6 mice/group). Equal variances were confirmed using the F‐test [F(5, 5) = 2.086, *p* = 0.4388]. The data were normally distributed; *p*‐values were calculated using the unpaired *t*‐test. **p < 0.01. (G) There was no difference CYP46A1 levels between the Ctrl and A/S groups (*n* = 6 mice/group). Equal variances were confirmed using the F‐test [F(5, 5) = 1.156, *p* = 0.8777]. The data were normally distributed; *p*‐values were calculated using the unpaired *t*‐test. (H) Representative immunofluorescence images of CYP46A1 (green) in the Ctrl and A/S groups (20× magnification; scale bar, 200 µm). (I) There was no difference in CYP46A1 intensities between the two groups (*n* = 6 mice/group). Equal variances were confirmed using the F‐test [F(5, 5) = 1.134, *p* = 0.8933]. The data were normally distributed; *p*‐values were calculated using the unpaired *t*‐test. Normally distributed data are presented as mean ± SD values; data that were not normally distributed are presented as median and interquartile range (IQR).

Neurons are responsible for cholesterol catabolism in the brain, and cholesterol is mainly converted into 24S‐hydroxycholesterol (24‐OHC) by cytochrome P450 46A1 (CYP46A1). Moreover, 24‐OHC can shuttle across the BBB and enter the peripheral circulation. Owing to the lack of an effective direct method for detecting brain cholesterol levels in clinical practice, we examined plasma 24‐OHC levels to indirectly assess the cholesterol levels in the brains of PND patients. The flowchart of the clinical study is presented in Figure 4A. Based on a postoperative MMSE score of ≤ 24, 15 patients were diagnosed with PND, and 45 patients were assigned to the NPND group. Table 1 summarizes the demographic characteristics of the patients. Although there was a statistical difference in MMSE scores of the NPND group before and after surgery [Preoperative MMSE score: the median was 28 points, and the interquartile range (IQR) was 1 point. Postoperative MMSE score: the median was 27 points, and the IQR was 2 points] (Mann–Whitney U test, *p *< 0.01, Figure 4B), the postoperative MMSE scores of all patients were higher than 24 points, the findings did not suggested **t**hat patients experienced cognitive decline after the surgery. The MMSE score of PND patients after surgery was significantly lower than that before surgery (Preoperative MMSE score: the median was 27 points and the IQR was 2 points. Postoperative MMSE score: the median was 22 points and the IQR was 2 points) (*t*‐test, *t*_28_ = 13, *p *< 0.01, Figure 4B). In addition, the postoperative MMSE score of PND patients was significantly lower than that of NPND patients after surgery (Mann–Whitney U test, *p *< 0.01, Figure 4B). Thus, the postoperative decline in MMSE scores was significantly greater in the PND group (the median was 5 points and the IQR was 3 points) than in the NPND group (the median was 1 point and the IQR was 1 point) (Mann–Whitney U test, *p *< 0.01, Figure 4B).

**TABLE 1:** Demographic characteristics of the NPND group and PND group.

Characteristics	NPND group (*n *= 45)	PND group (*n *= 15)	*p* value
Age (years)	71 (68, 75)	71 (71, 74)	0.384
Gender (male/female)	11/34	3/12	0.725
BMI	24.4 ± 2.2	25.3 ± 3.5	0.349
ASA grade			0.816
II	40	13	
III	5	2	
NYHA			0.059
I	2	3	
II	43	12	
Education duration (years)	10 (9, 11)	10 (8, 11)	0.894
Preoperative cholesterol (mmol/L)	4.42 ± 0.70	4.27 ± 0.64	0.467
Preoperative hemoglobin (g/L)	133.7 ± 12.5	129.0 ± 6.9	0.078
Duration of Surgery (min)	77 (71, 102)	76 (72, 103)	0.909

ASA: **American Society of Anesthesiologists;** NYHA: New York Heart Association Functional Classification.

Furthermore, there was no significant difference in 24‐OHC concentration before and after surgery in the NPND group (Pre‐operation: the median was 70.22 ng/mL and the IQR was 12.19 ng/mL. Post‐operation: the median was 66.75 ng/ml and the IQR was 5.73 ng/mL) (Mann–Whitney U test, *p *> 0.05, Figure 4C), whereas the plasma concentration of 24‐OHC after surgery was significantly lower than that before surgery in the PND group (Pre‐operation: the median was 72.05 ng/mL and the IQR was 14.98 ng/mL. Post‐operation: the median was 34.08 ng/mL and the IQR was 18.56 ng/mL) (*t*‐test, *t*_28_ = 10.26, *p *< 0.01, Figure 4C). In addition, the postoperative 24‐OHC concentration in PND patients was significantly lower than that of NPND patients (Mann–Whitney U test, *p *< 0.01, Figure 4C). Thus, the average concentration of 24‐OHC in the NPND group decreased by 2.44 ± 8.64 ng/mL after the operation, and the average concentration of 24‐OHC in the PND group decreased by 34.01 ± 8.73 ng/mL after surgery, which was statistically significant (*t*‐test, *t*_58 _= 12.23, *p *< 0.01, Figure 4C).

Similar results were observed in the mouse experiments (Figure 4D), anesthesia/surgery significantly downregulated the 24‐OHC levels in the plasma and in hippocampal tissues (plasma, *n* = 6 mice/group, *t*‐test, *t*_10 _= 6.662, *p *< 0.01, Figure 4E; hippocampal tissues, *n* = 6 mice/group, *t*‐test, *t*_10_ = 4.946, *p *< 0.01, Figure 4F). These results indicated that reduction of 24‐OHC in the peripheral circulation is positively correlated with the decrease in cholesterol content in the hippocampus.

Western blotting revealed that anesthesia/surgery did not significantly affect CYP46A1 level in the hippocampus (*n* = 6 mice/group, *t*‐test, *t*_10_ = 0.7175, *p *= 0.4895, Figure 4G). Immunofluorescence staining (Figure 4H) also showed no significant difference in CYP46A1 level in the hippocampal CA1 region between the two groups (n = 6 mice/group, *t*‐test, *t*_10 _= 0.034, *p *= 0.9735, Figure 4I). These preclinical and clinical findings suggested that anesthesia/surgery did not affect the ability of CYP46A1 to metabolize cholesterol and that the reduced 24‐OHC level in peripheral circulation may therefore be related to decreased cholesterol levels in the brain. Taken together, these findings indicate that 24‐OHC level can be regarded as a potential indicator of brain cholesterol levels.

### 2.5 Anesthesia/Surgery Impaired Synaptic Plasticity and Excitatory Synaptic Transmission in the Hippocampus

**FIGURE 5:**
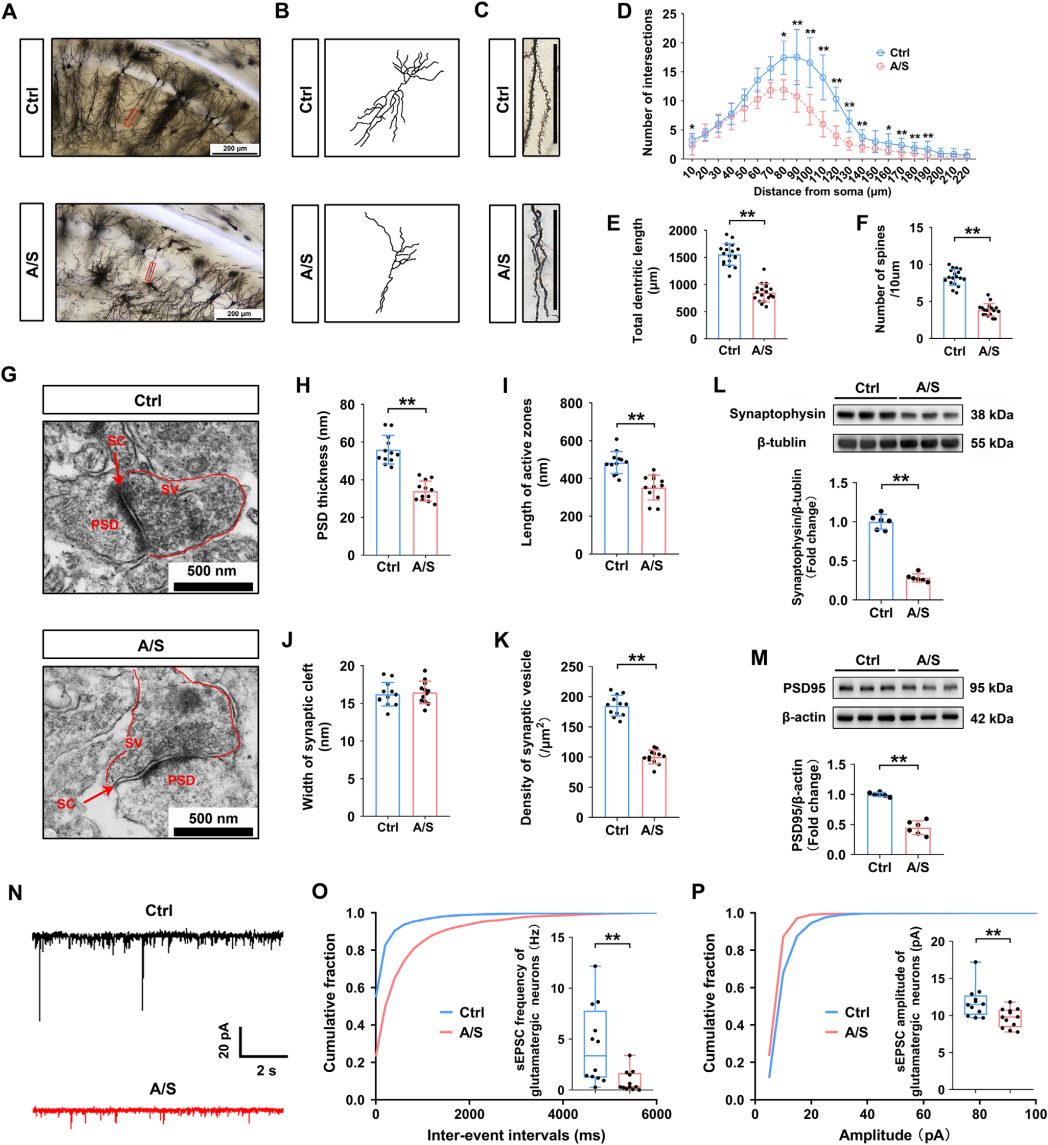
Anesthesia/surgery impaired synaptic plasticity and excitatory synaptic transmission in the dCA1. (A) Representative Golgi apparatus staining images in the Ctrl and A/S groups (20× magnification; scale bar, 200 µm). (B) Camera tracings of neurons. (C) Magnified view of dendritic spines (60× magnification; scale bar, 100 µm). 9D) Quantification of dendritic intersections (*n* = 18 neurons from 3 mice/group). The non‐normally distributed intersection data passed the normality test after logarithmic transformation (Y = Log(Y + 1)). Two‐way ANOVA was performed on the transformed data, followed by Bonferroni's post hoc test. (E) Quantitation of total dendritic length (*n* = 18 neurons from 3 mice/group). Equal variances were confirmed using the F‐test [F(17, 17) = 1.445, *p* = 0.4557]. The data were normally distributed; *p*‐values were calculated using the unpaired *t*‐test. (F) Quantitation of dendritic spine density (*n* = 18 neurons from 3 mice/group). Equal variances were confirmed using the F‐test [F(17, 17) = 1.72, *p* = 0.2735]. The data were normally distributed; *p*‐values were calculated using the unpaired *t*‐test. (G) Representative electron microscopy synaptic ultrastructure images in the Ctrl and A/S groups (scale bar, 500 nm). (H) Quantification of PSD thickness (*n* = 12 neurons from 3 mice/group). Equal variances were confirmed using the F‐test [F(11, 11) = 2.028, *p* = 0.2564]. The data were normally distributed; *p*‐values were calculated using the unpaired *t*‐test. (I) Quantitation of active zone length (*n* = 12 neurons from 3 mice/group). Equal variances were confirmed using the F‐test [F(11, 11) = 1.262, *p* = 0.7059]. The data were normally distributed; *p*‐values were calculated using the unpaired *t*‐test. (J) Quantitation of the synaptic cleft width (*n* = 12 neurons from 3 mice/group). Equal variances were confirmed using the F‐test [F(11, 11) = 1.119, *p* = 0.8549]. The data were normally distributed; *p*‐values were calculated using the unpaired *t*‐test. (K) Quantitation of presynaptic vesicle density (*n* = 12 neurons from 3 mice/group). Equal variances were confirmed using the F‐test [F(11, 11) = 2.251, *p* = 0.1941]. The data were normally distributed; *p*‐values were calculated using the unpaired *t*‐test. (L) Hippocampal synaptophysin levels (*n* = 6 mice/group). Equal variances were confirmed using the F‐test [F(5, 5) = 3.274, *p* = 0.219]. The data were normally distributed; *p*‐values were calculated using the unpaired *t*‐test. (M) Hippocampal PSD95 levels (*n* = 6 mice/group). Unequal variances were confirmed using the F‐test [F(5, 5) = 11.99, *p* = 0.0164]. The data were normally distributed; *p*‐values were calculated using Welch's *t*‐test. (N) Representative sEPSC of dCA1 glutamatergic neurons in the Ctrl and A/S groups; scale bars, 20 pA, 2 s. (O) sEPSC frequency in glutamatergic neurons was significantly reduced in the A/S group (*n* = 12 neurons from 6 mice/group). The data were not normally distributed; *p*‐values were calculated using the Mann–Whitney U test. (P) sEPSC amplitude in glutamatergic neurons was significantly lower in the A/S group (*n* = 12 neurons from 6 mice/group). The data were not normally distributed; *p*‐values were calculated using the Mann–Whitney U test. Normally distributed data are presented as mean ± SD values; data that were not normally distributed are presented as median and interquartile range (IQR). *p < 0.05, **p < 0.01 *vs* the Ctrl group.

Synaptic plasticity in the dCA1 was evaluated following the behavioral tests. Figure 5A,B shows the morphology of neurons and camera tracings in the dCA1 in the Ctrl and A/S groups. Figure 5C shows a magnified view of dendritic spines. Sholl analysis demonstrated that the number of dendritic intersections was significantly decreased in the A/S group compared with that in the Ctrl group (*n* = 18 neurons from 3 mice/group, two‐way ANOVA, F(1, 34) = 46.89, *p *< 0.01, Figure 5D). Total dendritic length (*n* = 18 neurons from 3 mice/group, *t*‐test, *t*_34 _= 11.58, *p *< 0.01, Figure 5E) and number of spines/10 µm (*n* = 18 neurons from 3 mice/group, *t*‐test, *t*_34 _= 13.64, *p *< 0.01, Figure 5F) were also significantly lower in the A/S group compared with those in the Ctrl group. The ultrastructure of the synapses observed using electron microscopy is shown in Figure 5G. Compared with those in the Ctrl group, postsynaptic density (PSD) thickness was significantly lower (*n* = 12 neurons from 3 mice/group, *t*‐test, *t*_22_ = 8.228, *p *< 0.01, Figure 5H), active zone length was shortened (*n* = 12 neurons from 3 mice/group, *t*‐test, *t*_22_ = 5.187, *p *< 0.01, Figure 5I), and presynaptic vesicle density (*n* = 12 neurons from 3 mice/group, *t*‐test, *t*_22_ = 14.36, *p *< 0.01, Figure 5K) was significantly reduced in the A/S group. Synaptic cleft width did not differ significantly between the two groups (*n* = 12 neurons from 3 mice/group, *t*‐test, *t*_22_ = 0.4006, *p* = 0.6926, Figure 5J). Correspondingly, the levels of synaptophysin and PSD95 in the hippocampus were significantly downregulated in the A/S group (n = 6 mice/group, *t*‐test, *t*_10 _= 16.17, *p *< 0.01, Figure 5L; *n* = 6 mice/group, Welch's *t*‐test, *t*_5.828_ = 11.36, *p *< 0.01; Figure 5M). These results suggested that cholesterol loss induced by surgery distinctly disturbed synaptic structural plasticity.

Given the importance of synaptic vesicles in the release of neurotransmitters, we tested the spontaneous excitatory postsynaptic current (sEPSC) of glutamatergic neurons in both groups following the behavioral tests (Figure 5N). sEPSC frequency and amplitude were significantly decreased in the A/S group (*n* = 12 neurons from 6 mice/group, Mann–Whitney U test, *p *< 0.01, Figure 5O; *n* = 12 neurons from 6 mice/group, Mann–Whitney U test, *p *< 0.01, Figure 5P), indicating a reduction in extrasynaptic excitatory transmission efficiency in glutamatergic neurons. This suggested that surgery‐induced cholesterol loss may inhibit transmission in excitatory neurons.

### 2.6 Supplementation of Cholesterol in the dCA1 Reversed the Reduced Long‐Term Potentiation (LTP) Caused by Anesthesia/Surgery

**FIGURE 6:**
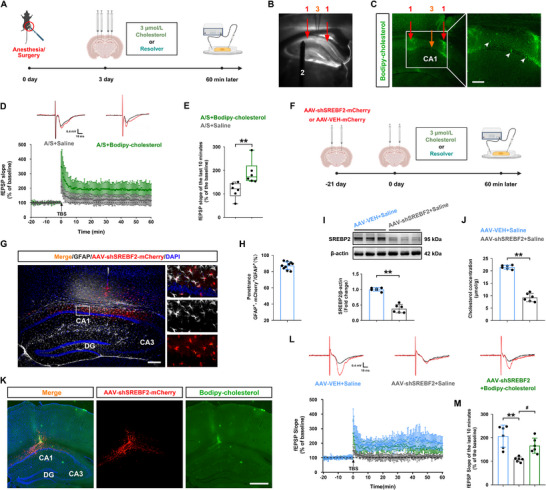
Cholesterol supplementation in the dCA1 rescued long‐term potentiation (LTP) in elderly mice that underwent anesthesia/surgery. (A) Flowchart of the experiments. (B) Diagrammatic representation of stimulation at the lateral Schaffer branch (location 2) and recording in the CA1 (location 3). The red arrow indicates the location of Bodipy‐cholesterol injection (location 1). (C) Representative immunofluorescence images of Bodipy‐cholesterol injection site (red arrow). The orange arrow indicates the location of the recording electrode, the white arrow head indicates binding of exogenous cholesterol to the cell membrane of pyramidal neurons. 10× magnification (left) and 20× magnification (right); Scale bar, 100 µm. (D) LTP recordings in the dCA1. The black arrow indicates the timing of the theta burst stimulation (TBS). (E) Average field excitatory postsynaptic potential (fEPSP) slope during the last 10 min after TBS (*n* = 6 mice/group). The data were not normally distributed; *p*‐values were calculated using the Mann–Whitney U test, **p < 0.01 *vs* the A/S+Saline group. (F) The flowchart of experiment. (G) Representative immunofluorescence images showed that AAV‐shSREBF2‐mCherry (red) was specifically co‐labeled with astrocyte marker GFAP (gray) in the dCA1 (10× **objective**; the scale bar is 200 µm). (H) AAV‐shSREBF2‐mCherry was mainly expressed in astrocytes, with high penetrance (>87% of the GFAP^+^ cells expressed mCherry) (*n* = 10 mice/group). (I) Quantitation of SREBP2 protein expression (*n* = 6 mice/group). Equal variances were confirmed by F‐test [F(5, 5) = 5.834, *p* = 0.0755]. The data follow a normal distribution, *p*‐values are calculated using unpaired *t*‐test. **p < 0.01 *vs* the AAV‐VEH+Saline group. (J) Cholesterol concentration in the hippocampus (*n* = 6 mice/group). Equal variances were confirmed by F‐test [F(5, 5) = 4.392, *p* = 0.1301]. The data follow a normal distribution, *p*‐values are calculated using unpaired *t*‐test. **p < 0.01 *vs* the AAV‐VEH+Saline group. (K) Representative immunofluorescence images of Bodipy‐cholesterol and AAV‐shSREBF2‐mCherry (4× **objective**; scale bar is 500 µm). (L) LTP recorded in the dCA1. (M) Average fEPSP slope during the last 10 min after TBS (*n* = 6 mice/group). Equal variances were confirmed by Brown‐Forsythe test [F(2, 15) = 3.13, *p* = 0.0731]. The data follow a normal distribution, *p*‐values are calculated using one‐way ANOVA with Tukey's post hoc test. Normally distributed data are presented as mean ± SD values; data that were not normally distributed are presented as median and interquartile range (IQR). **p < 0.01 *vs* the AAV‐VEH+Saline group; ^#^ *p *< 0.05 *vs* the AAV‐shSREBF2+Saline group. CA1: Cornu Ammonis 1; CA3: Cornu Ammonis 3; DG: Dentate Gyrus.

To clarify the correlation between hippocampal cholesterol deficiency and surgery‐induced synaptic plasticity impairment, we injected fluorescent Bodipy‐cholesterol or control solvent in the dendritic apical layer of the dCA1 area on the third day after surgery. Following the methods described in previous literature [22], we prepared brain slices and detected LTP 60 min later (Figure 6A). To avoid damaging the tissues in the recording area, the distance between the two injection points was at least 500 µm, and the evoked potential was recorded at a position between the two injection sites (Figure 6B). Figure 6C shows that fluorescent Bodipy‐cholesterol was accurately injected into the target area of the dCA1. Moreover, exogenous cholesterol is bound to the cell membrane of pyramidal neurons. Following TBS, the average fEPSP slope recorded in the dCA1 region was significantly higher in the A/S+Bodipy‐cholesterol group than in the A/S+Saline group (Figure 6D), a difference that was also sustained when analyzed specifically during the last 10 min of recording (*n* = 6 mice/group, Mann–Whitney U test, *p *< 0.01; Figure 6E).

To directly test whether cholesterol deficiency is the core downstream effector of SREBP2 dysregulation, we designed the experimental scheme outlined in Figure 6F. As shown in Figure 6G, AAV‐shSREBF2‐mCherry was mainly expressed in astrocytes with high penetrance (>87% of the GFAP cells expressed mCherry) (Figure 6H). Western blotting further revealed that hippocampal SREBP2 level was significantly reduced in the AAV‐shSREBF2+Saline group compared to that in the AAV‐VEH+Saline group (*n* = 6 mice/group, *t*‐test, *t*_10 _= 10.56, *p *< 0.01, Figure 6I). The content of cholesterol in hippocampal tissue was significantly decreased in the AAV‐shSREBF2 + Saline group (*n* = 6 mice/group, *t*‐test, *t*_10 _= 16.35, *p *< 0.01, Figure 6J). It indicated that astrocytic SREBF2 knockdown in the dCA1 region was successfully established.

Having confirmed that fluorescent Bodipy‐cholesterol was successfully delivered to dCA1 using the same stereotactic coordinates as the initial viral injection (Figure 6K), we evaluated its effects on LTP. Following TBS, the average fEPSP slope recorded in the dCA1 region was significantly lower in the AAV‐shSREBF2+Saline group than in the AAV‐VEH+Saline group; moreover, this impairment was fully rescued in the AAV‐shSREBF2+Bodipy‐cholesterol group (Figure 6L). Consistently, the average fEPSP slope during the last 10 min after TBS was also significantly reduced in the knockdown group and rescued by cholesterol supplementation (*n* = 6 mice/group, one‐way ANOVA, F(2, 15) = 13.15, *p *< 0.01, Figure 6M).

Collectively, our rescue experiments provided direct evidence that cholesterol supplementation reverses both surgery‐ and astrocytic SREBP2 knockdown‐induced LTP deficits, identifying local cholesterol deficiency as a key downstream pathogenic effector of astrocytic SREBP2 downregulation.

### 2.7 SREBP2 Overexpression in dCA1 Astrocytes Attenuated the Cognitive Dysfunction Caused by Anesthesia/Surgery

**FIGURE 7:**
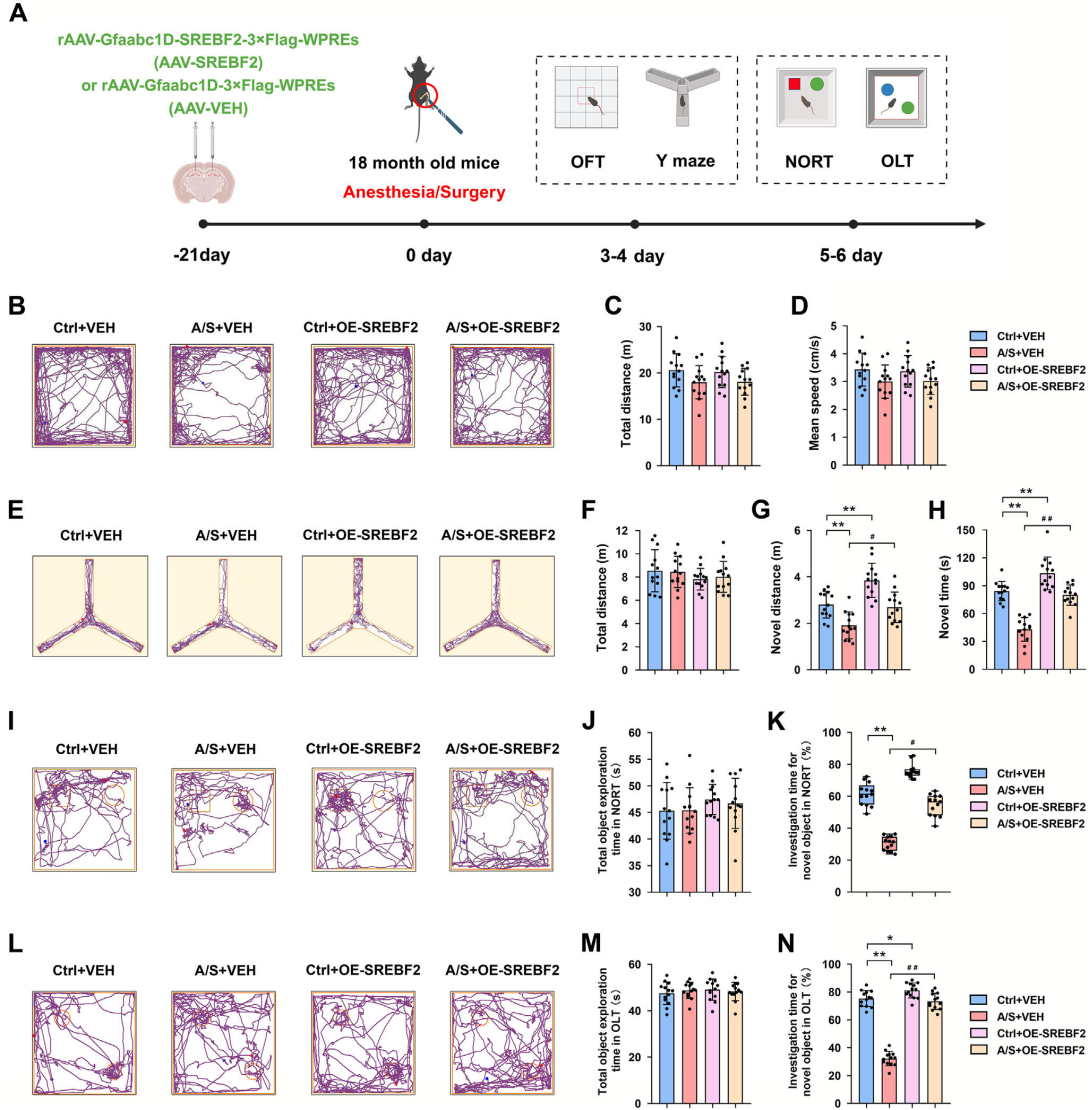
SREBP2 overexpression in dCA1 astrocytes attenuated the cognitive dysfunction caused by anesthesia/surgery. (A) Behavioral experiment timeline. (B) Representative trajectory in the OFT. (C) Comparison of the total moving distances among the four groups (*n* = 13 mice/group). Equal variances were confirmed using the Brown–Forsythe test [F(3, 48) = 0.1376, *p* = 0.937]. The data were normally distributed; *p*‐values were calculated using one‐way ANOVA with Tukey's post hoc test. (D) Comparison of the average speeds among the four groups (*n* = 13 mice/group). Equal variances were confirmed using the Brown–Forsythe test [F(3, 48) = 0.1376, *p* = 0.937]. The data were normally distributed; *p*‐values were calculated using one‐way ANOVA with Tukey's post hoc test. (E) Representative trajectory in the Y‐maze test. (F) Comparison of the total moving distances among four groups (*n* = 13 mice/group). Equal variances were confirmed using the Brown–Forsythe test [F(3, 48) = 1.729, *p* = 0.1736]. The data were normally distributed; *p*‐values were calculated using one‐way ANOVA with Tukey's post hoc test. (G) Comparison of the total moving distances in the novel arm among the four groups (*n* = 13 mice/group). Equal variances were confirmed using the Brown–Forsythe test [F(3, 48) = 0.2383, *p* = 0.8692]. The data were normally distributed; *p*‐values were calculated using one‐way ANOVA with Tukey's post hoc test. (H) Comparison of time in the novel arm among the four groups (*n* = 13 mice/group). Equal variances were confirmed using the Brown–Forsythe test [F(3, 48) = 1.003, *p* = 0.3996]. The data were normally distributed; *p*‐values were calculated using one‐way ANOVA with Tukey's post hoc test. (I) Representative trajectory in the NORT. (J) Comparison of total object exploration time among the four groups (*n* = 13 mice/group). Equal variances were confirmed using the Brown–Forsythe test [F(3, 48) = 1.242, *p* = 0.305]. The data were normally distributed; *p*‐values were calculated using one‐way ANOVA with Tukey's post hoc test. (K) Percentage of time spent exploring a novel object among the four groups (*n* = 13 mice/group). The data were not normally distributed; *p*‐values were calculated using Kruskal–Wallis test followed by Dunn's post hoc test. (L) Representative trajectory in the OLT. (M) Comparison of total object exploration time among the four groups (*n* = 13 mice/group). Equal variances were confirmed using the Brown–Forsythe test [F(3, 48) = 0.7107, *p* = 0.5504]. The data were normally distributed; *p*‐values were calculated using one‐way ANOVA with Tukey's post hoc test. (N) Percentage of time spent exploring a novel object among the four groups (*n* = 13 mice/group). Equal variances were confirmed using the Brown–Forsythe test [F(3, 48) = 0.2157, *p* = 0.885]. The data were normally distributed; *p*‐values were calculated using one‐way ANOVA with Tukey's post hoc test. Normally distributed data are presented as mean ± SD values; data that were not normally distributed are presented as median and interquartile range (IQR). *p < 0.05, **p < 0.01 *vs* the Ctrl+VEH group; ^#^ *p *< 0.05, ^##^ *p *< 0.01 *vs* the A/S+VEH group.

The behavioral experiment protocol is shown in Figure 7A. According to the movement trajectories in the four groups in Figure 7B, the mice showed similar total movement distances and movement speeds, indicating that surgical treatment and virus injection did not affect movement ability (*n* = 13 mice/group, one‐way ANOVA, F(3, 48) = 2.149, *p *= 0.1063, Figure 7C; *n* = 13 mice/group, one‐way ANOVA, F(3, 48) = 2.149, *p *= 0.1063, Figure 7D). The representative trajectories in the Y‐maze test (Figure 7E) showed no remarkable differences in total distance in exploring the three arms among the group (*n* = 13 mice/group, one‐way ANOVA, F(3, 48) = 0.795, *p *= 0.5027, Figure 7F), but A/S+OE‐SREBF2 group mice had longer movement distances and more time spent in the new arm than those in A/S+VEH group (*n* = 13 mice/group, one‐way ANOVA, F(3, 48) = 20.02, *p *< 0.01, Figure 7G; *n* = 13 mice/group, one‐way ANOVA, F(3, 48) = 47.01, *p* < 0.01, Figure 7H). The representative trajectories in the NORT (Figure 7I) showed that there was no significant differences in the time for exploring two objects among the groups (*n* = 13 mice/group, one‐way ANOVA, F(3, 48) = 0.7285, *p* = 0.54, Figure 7J); however, the time for exploring new objects in the A/S+OE‐SREBF2 group was significantly prolonged compared to that in the A/S+VEH group (*n* = 13 mice/group, Kruskal–Wallis test followed by Dunn's post hoc test, *p* < 0.01, Figure 7K). Similarly, in the OLT (Figure 7L), no remarkable difference was found in the time taken to explore two objects among the four groups (*n* = 13 mice/group, one‐way ANOVA, F(3, 48) = 0.3378, *p* = 0.798, Figure 7M), but the time taken to explore objects at new locations in the A/S+OE‐SREBF2 group was significantly restored compared with that in the A/S+VEH group (*n* = 13 mice/group, one‐way ANOVA, F(3, 48) = 214.3, *p* < 0.01, Figure 7N).

### 2.8 SREBP2 Overexpression in dCA1 Astrocytes Restored Cholesterol Synthesis in Elderly Mice That Underwent Anesthesia/Surgery

**FIGURE 8:**
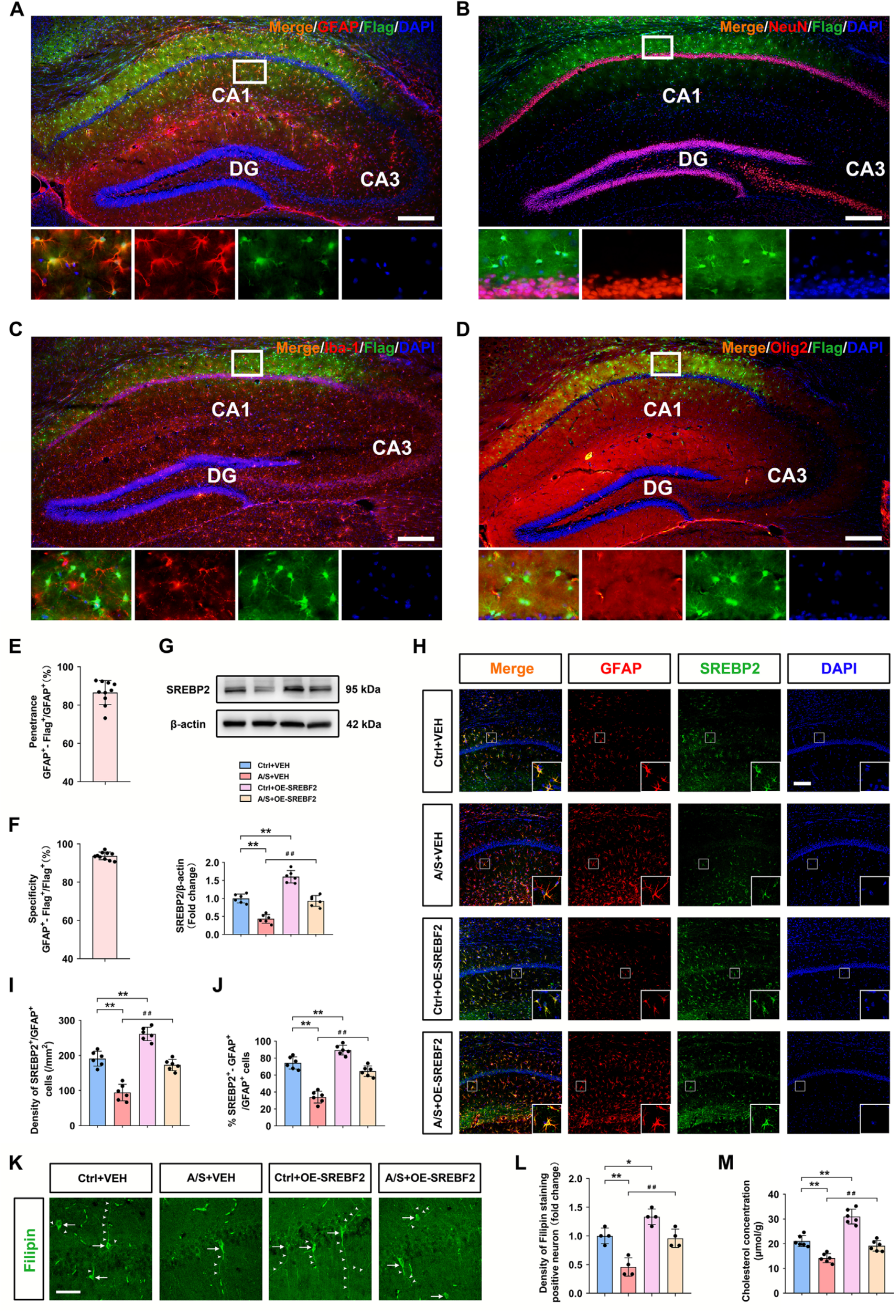
SREBP2 overexpression in dCA1 astrocytes restored cholesterol synthesis in elderly mice that underwent anesthesia/surgery. (A) Representative immunofluorescence images showed that rAAV‐SREBF2 with Flag (green) was specifically co‐labeled with the astrocyte marker GFAP (red) in the dCA1 (10× objective**;** the scale bar is 200 µm). (B) Representative immunofluorescence images of Flag (green) and neuron marker NeuN (red) (10× **objective**; the scale bar is 200 µm). (C) Representative immunofluorescence images of Flag (green) and microglial cell marker Iba‐1 (red) (10× **objective**; the scale bar is 200 µm). (D) Representative immunofluorescence images of Flag (green) and oligodendrocytes marker Olig2 (red) (10× **objective**; the scale bar is 200 µm). (E) Flag was mainly expressed in astrocytes with high penetrance (>86% of the GFAP cells expressed Flag) (*n* = 10 mice/group). (F) Flag was mainly expressed in astrocytes with high specificity (>93% Flag positive cells were also GFAP‐positive) (*n* = 10 mice/group). (G) Quantitation of SREBP2 levels (*n* = 6 mice/group). Equal variances were confirmed using the Brown–Forsythe test [F(3, 20) = 0.2891, *p* = 0.8327]. The data were normally distributed; *p*‐values were calculated using one‐way ANOVA with Tukey's post hoc test. (H) Representative immunofluorescence images of SREBP2 (green) and GFAP (red) in the four groups. Cells were counterstained with DAPI (blue) (20× **magnification**; scale bar, 100 µm). (I) Density of SREBP2/GFAP co‐labeled cells in the dCA1 (*n* = 6 mice/group). Equal variances were confirmed using the Brown–Forsythe test [F(3, 20) = 0.4763, *p* = 0.7023]. The data were normally distributed; *p*‐values were calculated using one‐way ANOVA with Tukey's post hoc test. (J) Co‐labeling rate of SREBP2^+^/GFAP^+^ cells in the dCA1 (*n* = 6 mice/group). Equal variances were confirmed using the Brown–Forsythe test [F(3, 20) = 0.238, *p* = 0.8688]. The data were normally distributed; *p*‐values were calculated using one‐way ANOVA with Tukey's post hoc test. (K) Representative immunofluorescence images of Filipin (green) in the four groups (60× magnification; scale bar, 100 µm). (L) Density of Filipin‐positive neurons in the dCA1 (*n* = 4 mice/group). Equal variances were confirmed using the Brown–Forsythe test [F(3, 12) = 0.2857, *p* = 0.8348]. The data were normally distributed; *p*‐values were calculated using one‐way ANOVA with Tukey's post hoc test. (M) Hippocampal cholesterol concentrations in the four groups (*n* = 6 mice/group). Equal variances were confirmed using the Brown–Forsythe test [F(3, 20) = 0.586, *p* = 0.6312]. The data were normally distributed; *p*‐values were calculated using one‐way ANOVA with Tukey's post hoc test. All data are presented as mean ± SD values; *p < 0.05, **p < 0.01 *vs* the Ctrl+VEH group; ^##^ *p *< 0.01 *vs* the A/S+VEH group. CA1: Cornu Ammonis 1; CA3: Cornu Ammonis 3; DG: Dentate Gyrus.

Confocal imaging revealed that rAAV‐SREBF2 overexpression with Flag was specifically co‐localized with the astrocyte marker GFAP in dCA1 cells (Figure 8A), the NeuN, Iba‐1, and Olig2 were not co‐labeled with Flag (Figure 8B–D). Within the transduced region, Flag was mainly expressed in astrocytes with high penetrance (>86% of the GFAP cells expressed Flag) (*n* = 10 mice/group, Figure 8E), and with high specificity (>93% Flag positive cells were also GFAP‐positive) (*n* = 10 mice/group, Figure 8F). Western blotting following the behavioral tests showed that the downregulation of SREBP2 levels in the hippocampus in the A/S+VEH group was rescued in the A/S+OE‐SREBF2 group (*n* = 6 mice/group, one‐way ANOVA, F(3, 20) = 66.71, *p* < 0.01, Figure 8G). Figure 8H shows representative GFAP/SREBP2 double immunofluorescence images, and the density and percentage of GFAP/SREBP2 double‐positive cells in dCA1 were significant lower in the A/S+VEH group compared with the Ctrl+VEH group, which were restored in the A/S+OE‐SREBF2 group (density: *n* = 6 mice/group, one‐way ANOVA, F(3, 20) = 69.04, *p *< 0.01, Figure 8I; percentage: *n* = 6 mice/group, one‐way ANOVA, F(3, 20) = 76.09, *p* < 0.01, Figure 8J). Filipin staining directly reflected the cholesterol content of neurons in each group (Figure 8K). The density of Filipin‐positive neurons in the dCA1 was significantly decreased in the A/S+VEH group compared to that in the Ctrl+VEH group, which was restored in the A/S+OE‐SREBF2 group (*n* = 4 mice/group, one‐way ANOVA, F(3, 12) = 23.68, *p *< 0.01, Figure 8L). The cholesterol content in the A/S+OE‐SREBF2 group was significantly higher than that in the A/S+VEH group (*n* = 6 mice/group, one‐way ANOVA, F(3, 20) = 54.45, *p *< 0.01, Figure 8M).

### 2.9 SREBP2 Overexpression in dCA1 Astrocytes Reversed Synaptic Plasticity Impairment and Excitatory Synaptic Transmission Inhibition Caused by Anesthesia/Surgery

**FIGURE 9:**
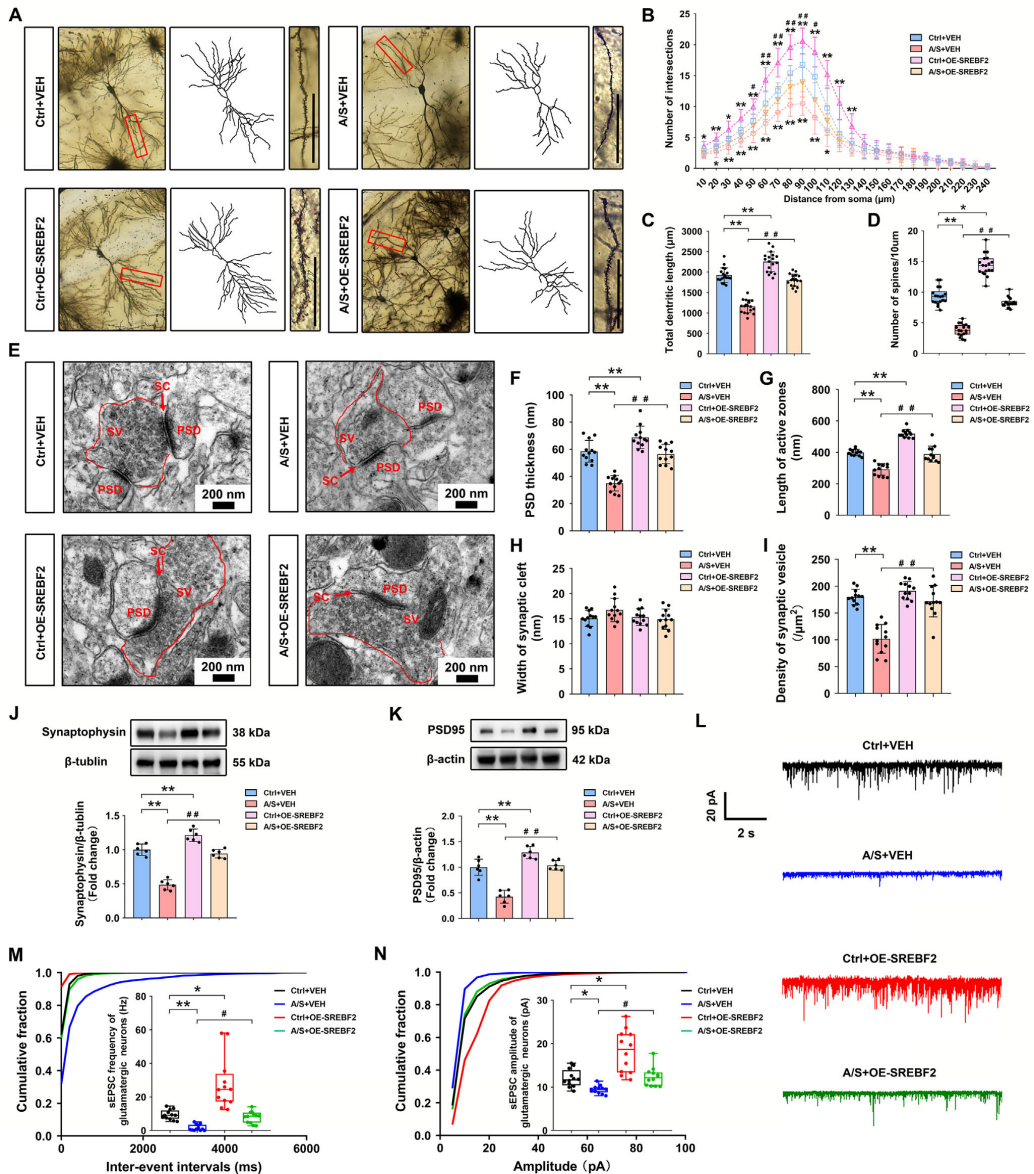
SREBP2 overexpression in dCA1 astrocytes reversed the synaptic plasticity impairment and excitatory synaptic transmission inhibition caused by anesthesia/surgery. (A) Representative Golgi apparatus staining images (20× **magnification**); camera tracings of neurons and magnified views of dendritic spines (60× magnification; scale bar, 100 µm) in the four groups. (B) Quantification of dendritic intersections (*n* = 18 neurons from 3 mice/group). The non‐normally distributed intersection data passed the normality test after undergoing the logarithmic transformation (Y = Log(Y + 1)); two‐way ANOVA was performed on the transformed data, followed by Bonferroni's post hoc test. (C) Quantitation of total dendritic length (*n* = 18 neurons from 3 mice/group). Equal variances were confirmed using the Brown–Forsythe test [F(3, 68) = 1.832, *p* = 0.1496]. The data were normally distributed; *p*‐values were calculated using one‐way ANOVA with Tukey's post hoc test. (D) Quantitation of dendritic spine density (*n* = 18 neurons from 3 mice/group). The data were non‐normally distributed; *p‐*values were calculated using the Kruskal–Wallis test followed by Dunn's post hoc test. (E) Representative synaptic ultrastructure electron microscopy images in the four groups (scale bar, 200 nm). (F) Quantification of PSD thickness (*n* = 12 neurons from 3 mice). Equal variances were confirmed using the Brown–Forsythe test [F(3, 44) = 0.3421, *p* = 0.795]. The data were normally distributed; *p*‐values were calculated using one‐way ANOVA with Tukey's post hoc test. (G) Quantitation of active zone length (*n* = 12 neurons from 3 mice/group). Equal variances were confirmed using the F‐test [F(3, 44) = 1.93, *p* = 0.1387]. The data were normally distributed; *p*‐values were calculated using one‐way ANOVA with Tukey's post hoc test. (H) Quantitation of synaptic cleft width (*n* = 12 neurons from 3 mice/group). Equal variances were confirmed using the Brown–Forsythe test [F(3, 44) = 0.529, *p* = 0.6647]. The data were normally distributed; *p*‐values were calculated using one‐way ANOVA with Tukey's post hoc test. (I) Quantitation of presynaptic vesicles density (*n* = 12 neurons from 3 mice/group). Equal variances were confirmed using the Brown–Forsythe test [F(3, 44) = 1.504, *p* = 0.2267]. The data were normally distributed; *p*‐values were calculated using one‐way ANOVA with Tukey's post hoc test. (J) Quantitation of synaptophysin levels (*n* = 6 mice/group). Equal variances were confirmed using the Brown–Forsythe test [F(3, 20) = 0.3298, *p* = 0.8039]. The data were normally distributed; *p*‐values were calculated using one‐way ANOVA with Tukey's post hoc test. (K) Quantitation of PSD95 levels (*n* = 6 mice/group). Equal variances were confirmed using the Brown–Forsythe test [F(3, 20) = 0.3308, *p* = 0.8031]. The data were normally distributed; *p*‐values were calculated using one‐way ANOVA with Tukey's post hoc test. (L) Representative sEPSC from dCA1 glutamatergic neurons in the four groups. Scale bar, 20 pA, 2 s. (M) Quantitation of the sEPSC frequency in glutamatergic neurons (*n* = 12 neurons from 6 mice/group). The data were not normally distributed; *p*‐values were calculated using Kruskal–Wallis test followed by Dunn's post hoc test. (N) Quantitation of sEPSC amplitude in glutamatergic neurons (*n* = 12 neurons from 6 mice/group). The data were not normally distributed; *p*‐values were calculated using Kruskal–Wallis test followed by Dunn's post hoc test. Normally distributed data are presented as mean ± SD values; data that were not normally distributed are presented as median and interquartile range (IQR). *p < 0.05, **p < 0.01 *vs* the Ctrl+VEH group; ^#^ *p *< 0.05, ^##^ *p *< 0.01 *vs* the A/S+VEH group.

Synaptic plasticity in the dCA1 was evaluated following the behavioral tests. Figure 9A shows the neuronal morphology, camera tracings, and a magnified view of dendritic spines of dCA1 neurons in the four groups. Compared with those in the A/S+VEH group, the number of intersections was significantly increased (*n* = 18 neurons from 3 mice/group, two‐way ANOVA, F(3, 68) = 30.97, *p *< 0.01, Figure 9B), total dendritic length became longer (*n* = 18 neurons from 3 mice/group, one‐way ANOVA, F(3, 68) = 110, *p *< 0.01, Figure 9C), and the density of dendritic spines was restored in the A/S+OE‐SREBF2 group (*n* = 18 neurons from 3 mice/group, Kruskal–Wallis test followed by Dunn's post hoc test, *p *< 0.01, Figure 9D). The synaptic ultrastructure in the four groups is shown in Figure 9E. Compared with those in the A/S+VEH group, PSD was significantly thickened (*n* = 12 neurons from 3 mice/group, one‐way ANOVA, F(3, 44) = 44.84, *p* < 0.01, Figure 9F), active zones became longer (*n* = 12 neurons from 3 mice/group, one‐way ANOVA, F(3, 44) = 82.26, *p* < 0.01, Figure 9G), and density of presynaptic vesicles was higher in the A/S+OE‐SREBF2 group (*n* = 12 neurons from 3 mice/group, one‐way ANOVA, F(3, 44) = 38.12, *p* < 0.01, Figure 9I). Synaptic cleft width did not differ among the four groups (*n* = 12 neurons from 3 mice/group, one‐way ANOVA, F(3, 44) = 2.356, *p *= 0.0847, Figure 9H). Meanwhile, synaptophysin and PSD95 levels in hippocampal tissue were found to be significantly higher in the A/S+OE‐SREBF2 group than in the A/S+VEH group (*n* = 6 mice/group, one‐way ANOVA, F(3, 20) = 90.7, *p *< 0.01, Figure 9J; *n* = 6 mice/group, one‐way ANOVA, F(3, 20) = 50.44, *p *< 0.01, Figure 9K). In conclusion, the synaptic plasticity impairment induced by anesthesia/surgery could be reversed by overexpressing SREBP2 in dCA1 astrocytes.

Representative sEPSC of dCA1 glutamatergic neurons are shown in Figure 9L. The electrophysiological data revealed a significant increase in sEPSC frequency and amplitude in the A/S+OE‐SREBF2 group than in the A/S+VEH group (*n* = 12 neurons from 6 mice/group, the data were not normally distributed, *p*‐values were calculated using Kruskal–Wallis test followed by Dunn's post hoc test, Figure 9M; *n* = 12 neurons from 6 mice/group, the data were not normally distributed, *p*‐values were calculated using Kruskal–Wallis test followed by Dunn's post hoc test, Figure 9N). These results demonstrated that the disordered cholesterol synthesis in astrocytes induced by the downregulation of SREBP2 plays a vital role in the synaptic plasticity impairment and excitatory synaptic transmission dysfunction caused by anesthesia/surgery.

### 2.10 Chemogenetic Inhibition of Astrocytes in the dCA1 Attenuated the Cognitive Impairment Caused by Anesthesia/Surgery

**FIGURE 10:**
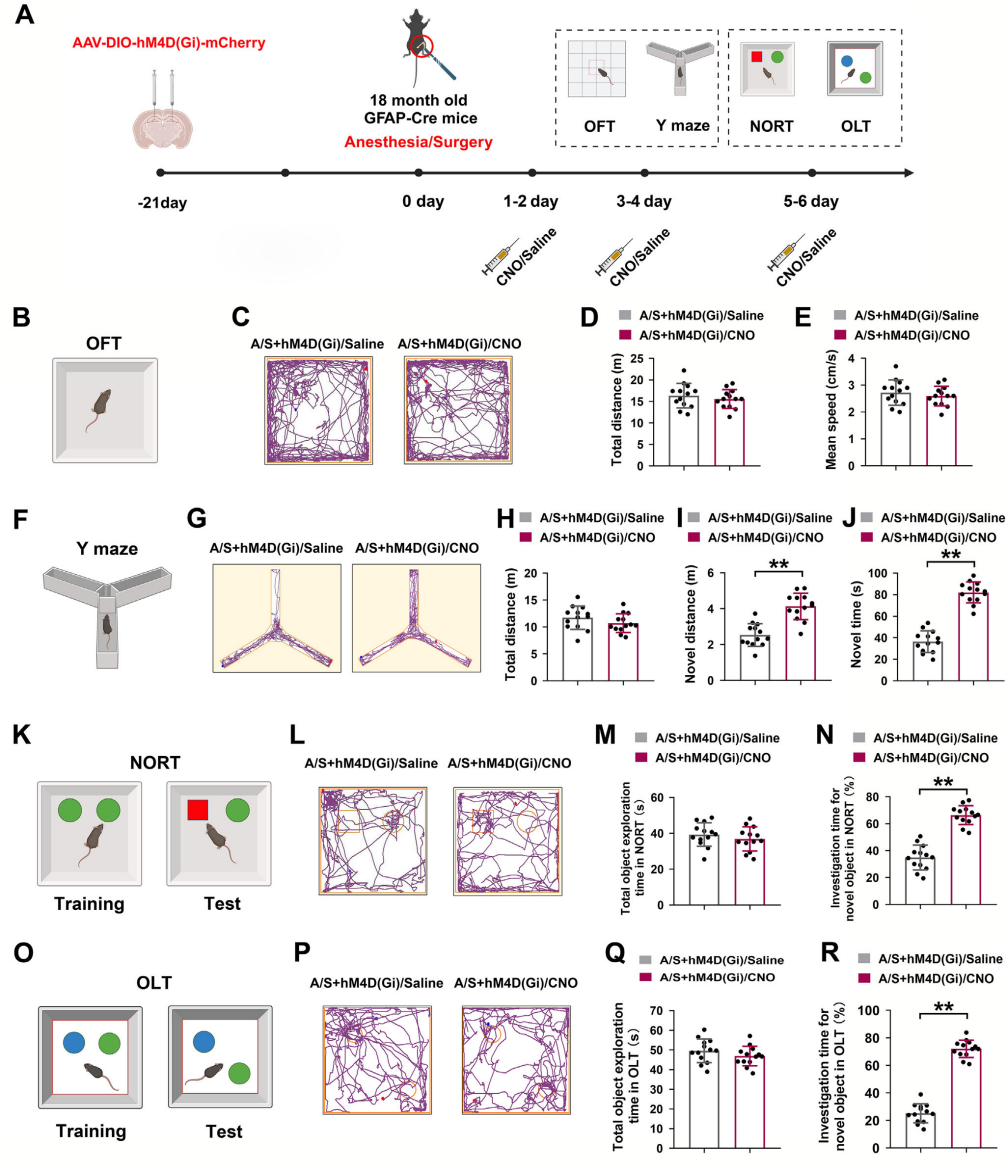
Chemogenetic inhibition of astrocytes in the dCA1 attenuated cognitive impairment caused by anesthesia/surgery. (A) The behavioral timeline and time points of CNO or saline injection. (B) Diagrammatic representation of the OFT. (C) Representative trajectory in the OFT. (D) Comparison of total moving distances between the two groups (*n* = 13 mice/group). Equal variances were confirmed using the F‐test [F(12,12) = 1.688, *p* = 0.377]. The data were normally distributed; *p*‐values were calculated using the unpaired *t*‐test. (E) Comparison of average speeds between the two groups (*n* = 13 mice/group). Equal variances were confirmed using the F‐test [F(12,12) = 1.688, *p* = 0.377]. The data were normally distributed; *p*‐values were calculated using the unpaired *t*‐test. (F) Diagrammatic representation of the Y‐maze test. (G) Representative trajectory in the Y‐maze test. (H) Comparison of total moving distances between the two groups (*n* = 13 mice/group). Equal variances were confirmed using the F‐test [F(12,12) = 1.539, *p* = 0.4663]. The data were normally distributed; *p*‐values were calculated using the unpaired *t*‐test. (I) Comparison of total moving distances in the novel arm between the two groups (*n* = 13 mice/group). Equal variances were confirmed using the F‐test [F(12,12) = 1.328, *p* = 0.631]. The data were normally distributed; *p*‐values were calculated using the unpaired *t*‐test. (J) Comparison of time spent in the novel arm between the two groups (*n* = 13 mice/group). Equal variances were confirmed using the F‐test [F(12,12) = 1.085, *p* = 0.8899]. The data were normally distributed; *p*‐values were calculated using the unpaired *t*‐test. (K) Diagrammatic representation of the NORT. (L) Representative trajectory in the NORT. (M) Comparison of total object exploration time between the two groups (*n* = 13 mice/group). Equal variances were confirmed using the F‐test [F(12,12) = 1.075, *p* = 0.9022]. The data were normally distributed; *p*‐values were calculated using the unpaired *t*‐test. (N) Percentage of time spent exploring a novel object (*n* = 13 mice/group). Equal variances were confirmed using the F‐test [F(12,12) = 1.729, *p* = 0.3556]. The data were normally distributed; *p*‐values were calculated using the unpaired *t*‐test. (O) Diagrammatic representation of the OLT. (P) Representative trajectory in the OLT. (Q) Comparison of total object exploration time between the two groups (*n* = 13 mice/group). Equal variances were confirmed using the F‐test [F(12,12) = 1.46, *p* = 0.5223]. The data were normally distributed; *p*‐values were calculated using the unpaired *t*‐test. (R) Percentage of time spent exploring a novel object (*n* = 13 mice/group). Equal variances were confirmed using the F‐test [F(12,12) = 1.208, *p* = 0.7484]. The data were normally distributed; *p*‐values were calculated using the unpaired *t*‐test. All data are presented as mean ± SD values. **p < 0.01 *vs* the A/S+hM4D(Gi)/Saline group.

To inhibit reactive astrocytes, a chemogenetic virus was injected into the bilateral dCA1 region of GFAP‐Cre mice 21 days before surgery. The flowchart of the behavioral tests and CNO injection strategy is shown in Figure 10A. CNO, a ligand for the chemically engineered receptor, was injected intraperitoneally from postoperative days 1 to 6 to inhibit astrocyte activation, and behavioral tests were conducted 30 min after injection. The movement trajectories in the OFT are shown in Figure 10C. There were no remarkable differences in total movement distance or mean movement speed between the two groups (*n* = 13 mice/group, *t*‐test, *t*_24_ = 0.7902, *p *= 0.4372, Figure 10D; *n* = 13 mice/group, *t*‐test, *t*_24_ = 0.7902, *p* = 0.4372, Figure 10E). Movement trajectories in the Y‐maze are shown in Figure 10G. Although there was no significant difference in the total movement distance between the two groups (*n* = 13 mice/group, *t*‐test, *t*_24_ = 1.315, *p* = 0.201, Figure 10H), mice in the A/S+hM4D(Gi)/CNO group had a longer exploration distance and time in the new arm than those in the A/S+hM4D(Gi)/Saline group (*n* = 13 mice/group, *t*‐test, *t*_24_ = 5.897, *p *< 0.01, Figure 10I; *n* = 13 mice/group, *t*‐test, *t*_24_ = 11.72, *p* < 0.01, Figure 10J). The movement trajectories in the NORT are shown in Figure 10L. No significant difference was found in the total object exploration time between the two groups (*n* = 13 mice/group, *t*‐test, *t*_24_ = 0.932, *p* = 0.3606, Figure 10M). However, the percentage of investigation time for a novel object was significantly higher in the A/S+hM4D(Gi)/CNO group than in the A/S+hM4D(Gi)/Saline group (*n* = 13 mice/group, *t*‐test, *t*_24_ = 9.802, *p *< 0.01, Figure 10N). The movement trajectories in the OLT are shown in Figure 10P, and no significant difference was found in the total object exploration time between the two groups (*n* = 13 mice/group, *t*‐test, *t*_24_ = 1.249, *p *= 0.2237, Figure 10Q). However, the percentage of time spent exploring an object in a new location was significantly higher in the A/S+hM4D(Gi)/CNO group than in the A/S+hM4D(Gi)/Saline group (*n* = 13 mice/group, *t*‐test, *t*_24_ = 18.09, *p *< 0.01, Figure 10R).

### 2.11 Inhibiting Reactive Astrocytes in the dCA1 Rescued Cholesterol Synthesis of Elderly Mice Under Anesthesia/Surgery

**FIGURE 11:**
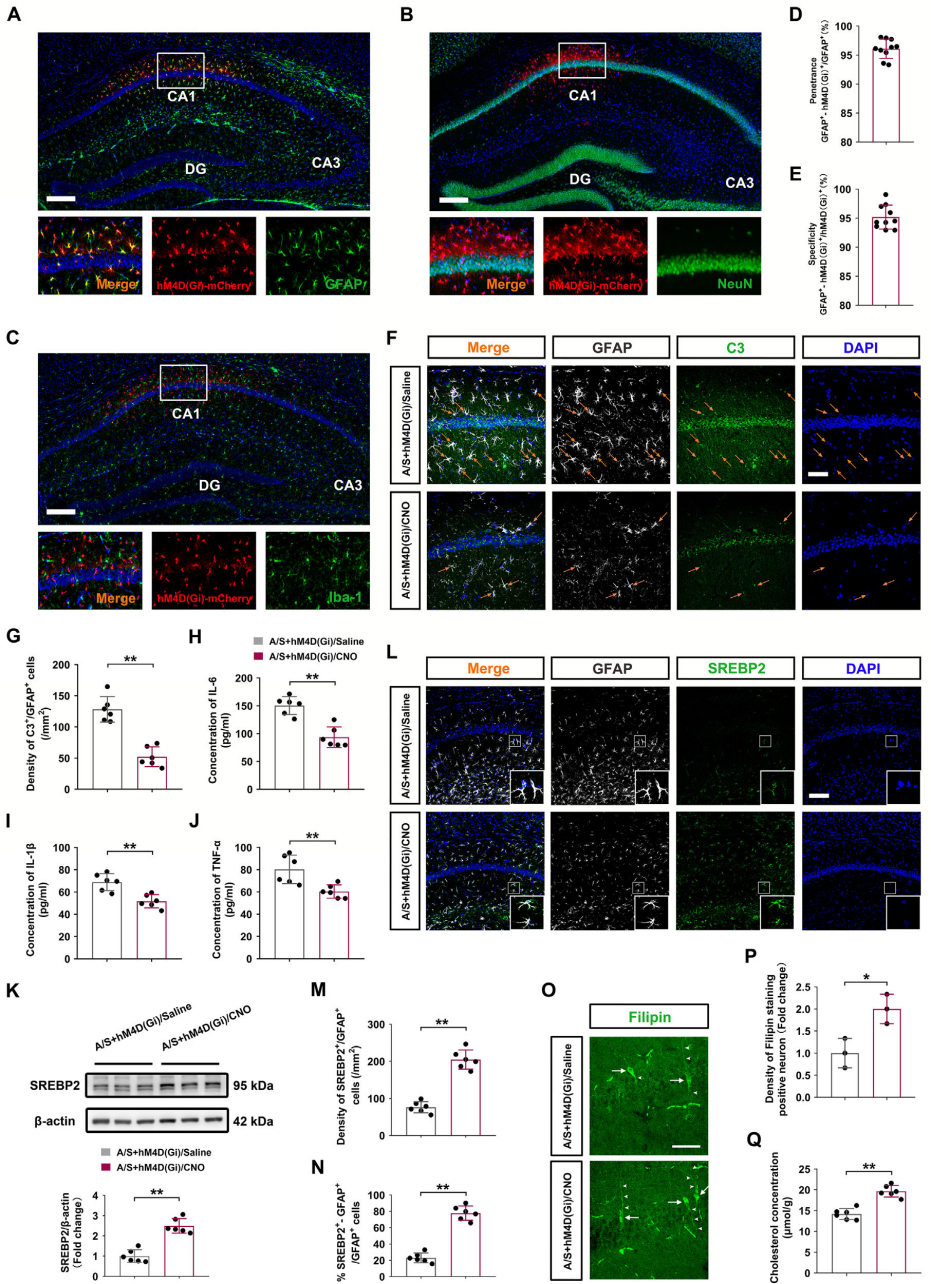
Chemogenetic inhibition of astrocytes in the dCA1 rescued cholesterol synthesis in elderly mice that underwent anesthesia/surgery. (A) Representative immunofluorescence images of hM4D(Gi)‐mCherry (red) and the astrocyte marker GFAP (green) (scale bar, 200 µm). (B) Representative immunofluorescence images of hM4D(Gi)‐mCherry (red) and the neuronal marker NeuN (green) (scale bar, 200 µm). (C) Representative immunofluorescence images of hM4D(Gi)‐mCherry (red) and the microglial marker Iba‐1 (green) (scale bar, 200 µm). (D) hM4D(Gi)‐mCherry was mainly expressed in astrocytes with high penetrance (>96% of the GFAP cells expressed mCherry) (*n* = 10 mice/group). (E) hM4D(Gi)‐mCherry was mainly expressed in astrocytes with high specificity (>95% mCherry positive cells were also GFAP‐positive). No co‐localization of hM4D(Gi)‐mCherry with NeuN or Iba1 was detected (*n* = 10 mice/group). (F) Representative immunofluorescence images of C3 (green) and GFAP (gray); cells were counterstained with DAPI (blue); the orange arrow indicates the double‐positive cells (40× magnification; scale bar, 50 µm). (G) Density of C3/GFAP co‐labeled cells in the dCA1 (*n* = 6 mice/group). Equal variances were confirmed using the F‐test [F(5,5) = 1.684, *p* = 0.5812]. The data followed a normal distribution; *p*‐values were calculated using the unpaired *t*‐test. (H) Analysis of IL‐6 levels in the dCA1 (*n* = 6 mice/group). Equal variances were confirmed by F‐test [F(5,5) = 1.294, *p* = 0.784]. The data were normally distributed; *p*‐values were calculated using the unpaired *t*‐test. (I) Analysis of IL‐1β levels in the dCA1 (*n* = 6 mice/group). Equal variances were confirmed using the F‐test [F(5,5) = 1.668, *p* = 0.588]. The data were normally distributed; *p*‐values were calculated using the unpaired *t*‐test. (J) Analysis of TNF‐α levels in the dCA1 (*n* = 6 mice/group). Equal variances were confirmed using the F‐test [F(5,5) = 4.579, *p* = 0.1204]. The data were normally distributed; *p*‐values were calculated using the unpaired *t*‐test. (K) Comparison of SREBP2 protein expression between the A/S+hM4D(Gi)/Saline and A/S+hM4D(Gi)/CNO groups (*n* = 6 mice/group). Equal variances were confirmed using the F‐test [F(5,5) = 1.308, *p* = 0.7757]. The data were normally distributed; *p*‐values were calculated using the unpaired *t*‐test. (L) Representative immunofluorescence images of SREBP2 (green) and GFAP (gray); cells were counterstained with DAPI (blue) (20× magnification; scale bar, 100 µm). (M) Density of SREBP2/GFAP co‐labeled cells in the dCA1 (*n* = 6 mice/group). Equal variances were confirmed using the F‐test [F(5,5) = 2.899, *p* = 0.2677]. The data were normally distributed; *p*‐values were calculated using the unpaired *t*‐test. (N) Co‐labeling rate of SREBP2^+^/GFAP^+^ cells in the dCA1 (*n* = 6 mice/group). Equal variances were confirmed using the F‐test [F(5,5) = 2.183, *p* = 0.4116]. The data were normally distributed; *p*‐values were calculated using the unpaired *t*‐test. (O) Representative immunofluorescence images of Filipin (green) (60× magnification; scale bar, 100 µm). (P) Density of Filipin‐positive neurons in the dCA1 (*n* = 3 mice/group). Equal variances were confirmed using the F‐test [F(2,2) = 1]. The data were normally distributed; *p*‐values were calculated using the unpaired *t*‐test. (Q) Cholesterol concentrations in the hippocampus (*n* = 6 mice/group). Equal variances were confirmed using the F‐test [F(5,5) = 1.102, *p* = 0.918]. The data were normally distributed; *p*‐values were calculated using the unpaired *t*‐test. All data are presented as mean ± SD values. *p < 0.05, **p < 0.01 *vs* the A/S+hM4D(Gi)/Saline group. CA1: Cornu Ammonis 1; CA3: Cornu Ammonis 3; DG: Dentate Gyrus.

Immunofluorescence staining (Figure 11A–C) revealed that GFAP was almost entirely co‐labeled with hM4D(Gi)‐mCherry in the dCA1, while NeuN and Iba‐1 were not co‐labeled with hM4D(Gi)‐mCherry. Within the transduced region, hM4D(Gi) was mainly expressed in astrocytes with high penetrance (>96% of the GFAP cells expressed mCherry signal) (*n* = 10 mice/group, Figure 11D), and with high specificity (>95% mCherry‐positive cells were also GFAP‐positive) (*n* = 10 mice/group, Figure 11E). Complement C3 is regarded as a key marker of A1 phenotypic reactive astrocytes, we examined the hippocampal C3^+^/GFAP^+^ cells in dCA1 using immunofluorescence staining (Figure 11F). The number of C3^+^ **/**GFAP^+^ cells in the dCA1 was significantly lower in the A/S+hM4D(Gi)/CNO group than the A/S+hM4D(Gi)/Saline group (*n* = 6 mice/group, *t*‐test, *t*_10_ = 7.174, *p *< 0.01, Figure 11G). Furthermore, levels of pro‐inflammatory cytokines (IL‐6, IL‐1β, TNF‐α) were notably reduced in the A/S+hM4D(Gi)/CNO group (IL‐6: *n* = 6 mice/group, *t*‐test, t_10_ = 5.666, *p *< 0.01; IL‐1β: *n* = 6 mice/group, t‐test, t_10_ = 4.359, *p *< 0.01; TNF‐α: *n* = 6 mice/group, *t*‐test, t_10_ = 3.44, *p* < 0.01; Figure 11H–J). These findings indicated that chemogenetic inhibition of astrocytes alleviated hippocampal neuroinflammation induced by surgery.

Next, we assessed SREBP2 expression in dCA1 astrocytes. Western blotting analysis revealed a significant increase in SREBP2 level in the A/S+hM4D(Gi)/CNO group compared to that in the A/S+hM4D(Gi)/Saline group (*n* = 6 mice/group, *t*‐test, *t*_10_ = 7.658, *p* < 0.01, Figure 11K). Immunofluorescence (Figure 11L) showed that SREBP2 level in dCA1 astrocytes was significantly higher in the A/S+hM4D(Gi)/CNO group (density: *n* = 6 mice/group, *t*‐test, *t*_10_ = 10.53, *p *< 0.01, Figure 11M. Percentage: *n* = 6 mice/group, *t*‐test, *t*_10_ = 12.69, *p *< 0.01, Figure 11N). Moreover, the density of Filipin‐positive neurons in the dCA1 was significantly higher in the A/S+hM4D(Gi)/CNO group than in the A/S+hM4D(Gi)/Saline group (*n* = 3 mice/group, *t*‐test, *t*_4_ = 3.674, *p *= 0.0213, Figure 11O,P). The results revealed that the cholesterol content of the hippocampus in the A/S+hM4D(Gi)/CNO group was significantly higher than that in the A/S+hM4D(Gi)/Saline group (*n* = 6 mice/group, *t*‐test, *t*_10_ = 7.074, *p *< 0.01, Figure 11Q). In conclusion, impaired cholesterol synthesis, mediated by the downregulation of SREBP2, is a core pathological feature of reactive astrocytes after surgery.

### 2.12 Chemogenetic Inhibition of Astrocytes in the dCA1 Reversed Anesthesia/Surgery‐Induced Synaptic Plasticity Impairment and Excitatory Synaptic Transmission Inhibition

**FIGURE 12:**
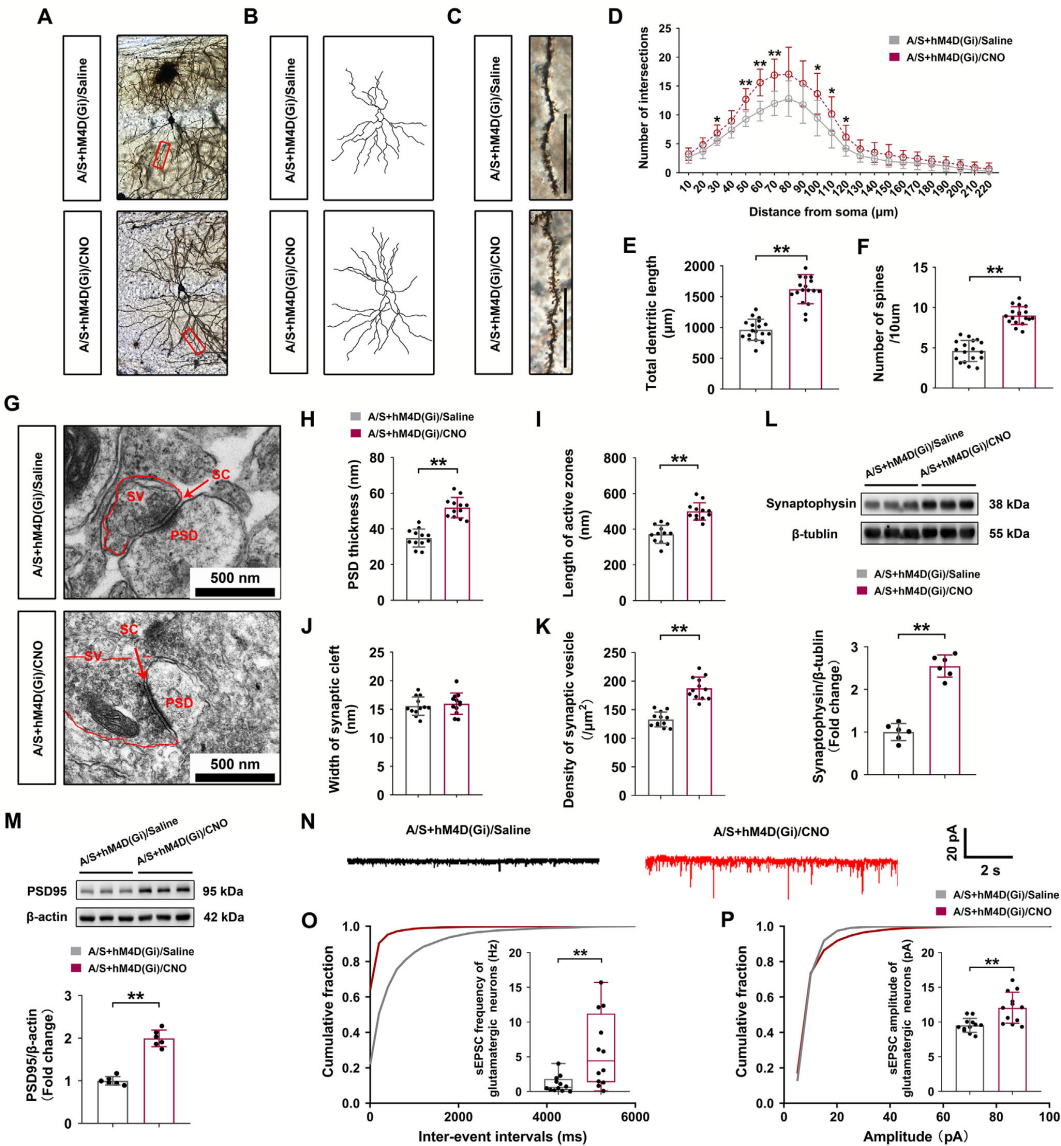
Chemogenetic inhibition of astrocytes in the dCA1 reversed synaptic plasticity impairment and excitatory synaptic transmission inhibition caused by anesthesia/surgery. (A) Representative Golgi apparatus staining images in the A/S+hM4D(Gi)/Saline and A/S+hM4D(Gi)/CNO groups (20× **magnification**). (B) Camera tracings of neurons. (C) Magnified view of dendritic spines (60× magnification; scale bar, 100 µm). (D) Quantification of dendritic intersections (*n* = 18 neurons from 3 mice/group). The non‐normal distributed intersection data passed the normality test after undergoing the logarithmic transformation (Y = Log(Y + 1)); two‐way ANOVA was performed on the transformed data, followed by Bonferroni's post hoc test. (E) Quantitation of the total dendritic length (*n* = 18 neurons from 3 mice/group). Equal variances were confirmed using the F‐test [F(17,17) = 1.933, *p* = 0.1845]. The data were normally distributed; *p*‐values were calculated using the unpaired *t*‐test. (F) Quantitation of the dendritic spine density (*n* = 18 neurons from 3 mice/group). Equal variances were confirmed using the F‐test [F(17,17) = 1.431, *p* = 0.4678]. The data were normally distributed; *p*‐values were calculated using the unpaired *t*‐test. (G) Representative synaptic ultrastructure electron microscopy images (scale bar, 500 nm). (H) Quantification of PSD thickness (*n* = 12 neurons from 3 mice/group). Equal variances were confirmed using the F‐test [F(11,11) = 1.28, *p* = 0.6894]. The data were normally distributed; *p*‐values were calculated using the unpaired *t*‐test. (I) Quantitation of active zone length (*n* = 12 neurons from 3 mice/group). Equal variances were confirmed using the F‐test [F(11,11) = 1.074, *p* = 0.9076]. The data were normally distributed; *p*‐values were calculated using the unpaired *t*‐test. (J) Quantitation of the synaptic cleft width (*n* = 12 neurons from 3 mice/group). Equal variances were confirmed using the F‐test [F(11,11) = 1.361, *p* = 0.6178]. The data were normally distributed; *p*‐values were calculated using the unpaired *t*‐test. (K) Quantitation of presynaptic vesicles density (*n* = 12 neurons from 3 mice/group). Equal variances were confirmed using the F‐test [F(11,11) = 2.179, *p* = 0.2122]. The data were normally distributed; *p*‐values were calculated using the unpaired *t*‐test. (L) Levels of synaptophysin in the hippocampus (*n* = 6 mice/group). Equal variances were confirmed using the F‐test [F(5,5) = 1.707, *p* = 0.5717]. The data were normally distributed; *p*‐values were calculated using the unpaired *t*‐test. M) Levels of PSD95 in the hippocampus (*n* = 6 mice/group). Equal variances were confirmed using the F‐test [F(5,5) = 3.896, *p* = 0.1618]. The data were normally distributed; *p*‐values were calculated using the unpaired *t*‐test. (N) Representative sEPSC from dorsal hippocampal glutamatergic neurons. Scale bars, 20 pA, 2 s. (O) Quantitation of sEPSC frequency in glutamatergic neurons (*n* = 12 neurons from 6 mice/group). The data were not normally distributed; *p*‐values were calculated using the Mann–Whitney U test. (P) Quantitation of sEPSC amplitude in glutamatergic neurons (*n* = 12 neurons from 6 mice/group). Unequal variances were confirmed using the F‐test [F(11,11) = 4.902, *p* = 0.0139]. The data were normally distributed; *p*‐values were calculated using Welch's *t*‐test. Normally distributed data are presented as mean ± SD values; data that were not normally distributed are presented as median and interquartile range (IQR). *p < 0.05, **p < 0.01 *vs* the A/S+hM4D(Gi)/Saline group.

Synaptic plasticity in the dCA1 was evaluated following the behavioral tests. Figure 12A,B shows the morphology of neurons and camera tracings in the two groups, and Figure 12C shows a magnified view of dendritic spines. Compared with those in the A/S+hM4D(Gi)/Saline group, the number of intersections was significantly increased (*n* = 18 neurons from 3 mice/group, two‐way ANOVA, F(1, 34) = 24.04, *p *< 0.01, Figure 12D), the total dendritic length was longer (*n* = 18 neurons from 3 mice/group, *t*‐test, *t*_34_ = 9.57, *p *< 0.01, Figure 12E), and the density of dendritic spines was restored in the A/S+hM4D(Gi)/CNO group (*n* = 18 neurons from 3 mice/group, *t*‐test, *t*_34_ = 10.93, *p *< 0.01, Figure 12F). The representative electron microscopy images are shown in Figure 12G. Compared with those in the A/S+hM4D(Gi)/Saline group, PSD was significantly thickened (*n* = 12 neurons from 3 mice/group, *t*‐test, *t*_22_ = 7.747, *p *< 0.01, Figure 12H), active zones were longer (*n* = 12 neurons from 3 mice/group, *t*‐test, *t*_22_ = 6.361, *p *< 0.01, Figure 12I), and the density of presynaptic vesicles was higher in the A/S+hM4D(Gi)/CNO group (*n* = 12 neurons from 3 mice/group, *t*‐test, *t*_22_ = 8.124, *p *< 0.01, Figure 12K). Synaptic cleft width did not differ between the two groups (*n* = 12 neurons from 3 mice/group, *t*‐test, *t*_22_ = 0.5949, *p* = 0.558, Figure 12J). Synaptophysin and PSD95 levels in hippocampal tissue were significantly higher in the A/S+hM4D(Gi)/CNO group than in the A/S+hM4D(Gi)/Saline group (*n* = 6 mice/group, *t*‐test, *t*_10_ = 11.49, *p* < 0.01, Figure 12L; *n* = 6 mice/group, *t*‐test, *t*_10_ = 11.04, *p* < 0.01, Figure 12M). Representative sEPSC of dCA1 glutamatergic neurons are shown in Figure 12N. The electrophysiological data revealed that sEPSC frequency and amplitude were significantly higher in the A/S+hM4D(Gi)/CNO group than in the A/S+hM4D(Gi)/Saline group (*n* = 12 neurons from 6 mice/group, Mann–Whitney U test, *p* < 0.01, Figure 12O; *n* = 12 neurons from 6 mice/group, Welch's *t*‐test, *t*_15.31_ = 3.595, *p* < 0.01, Figure 12P). Collectively, these findings suggested that inhibiting reactive astrocytes in the dCA1 to salvage cholesterol synthesis could reverse the deleterious effects on synaptic plasticity and excitatory synaptic transmission induced by anesthesia/surgery.

### 2.13 Minocycline Restored SREBP2‐Mediated Cholesterol Homeostasis in Astrocytes by Suppressing Neuroinflammation

**FIGURE 13:**
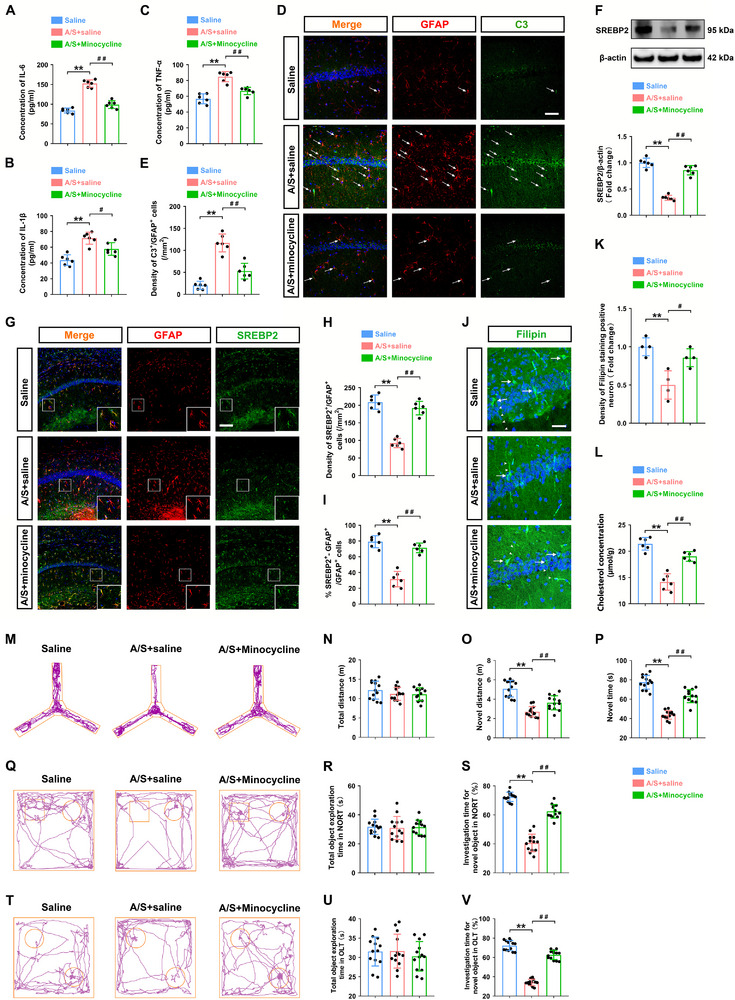
Anesthesia/surgery impaired cognitive function, increased pro‐inflammatory factor levels, and suppressed astrocytic SREBP2 levels in aged mice. (A–C) Hippocampal levels of IL‐6, IL‐1β, and TNF‐α 3 days after surgery (*n* = 6 mice/group). Equal variances were confirmed using the Brown–Forsythe test; IL‐6: [F(2, 15) = 0.66, *p* = 0.5312]; IL‐1β: [F(2, 15) = 0.02894, *p* = 0.9715]; TNF‐α: [F(2, 15) = 0.4066, *p* = 0.673]. The data were normally distributed; *p*‐values were calculated using one‐way ANOVA with Tukey's post hoc test. (D) Representative confocal images staining for C3 (green) and GFAP (red) in the dCA1. Cells were counterstained with DAPI (blue); the white arrow indicates the double‐positive cells (40× magnification; scale bar, 50 µm). (E) Density of C3/GFAP co‐labeled cells (*n* = 6 mice/group). Equal variances were confirmed using the Brown–Forsythe test [F(2, 15) = 0.8925, *p* = 0.4303]. The data were normally distributed; *p*‐values were calculated using one‐way ANOVA with Tukey's post hoc test. (F) Comparison of SREBP2 protein expression among three groups (*n* = 6 mice/group). Equal variances were confirmed by Brown‐Forsythe test [F(2, 15) = 0.981, *p* = 0.3978]. The data follow a normal distribution, *p*‐values are calculated using one‐way ANOVA with Tukey's post hoc test. (G) Representative immunofluorescence images of SREBP2 (green) and GFAP (red) in the dCA1; cells were counterstained with DAPI (blue) (20× magnification; scale bar, 100 µm). (H) Density of SREBP2/GFAP co‐labeled cells (*n* = 6 mice/group). Equal variances were confirmed using the Brown–Forsythe test [F(2, 15) = 0.557, *p* = 0.5843]. The data were normally distributed; *p*‐values were calculated using one‐way ANOVA with Tukey's post hoc test. (I) Co‐labeling rate of SREBP2^+^/GFAP^+^ cells (*n* = 6 mice per group). Equal variances were confirmed using the Brown–Forsythe test [F(2, 15) = 0.6318, *p* = 0.5452]. The data were normally distributed; *p*‐values were calculated using one‐way ANOVA with Tukey's post hoc test. (J) Representative immunofluorescence images of Filipin (green) in the dCA1 (60× magnification; scale bar, 100 µm). (K) Density of Filipin‐positive neurons in dCA1 (*n* = 4 mice/group). Brown–Forsythe test [F(2, 9) = 1, *p* = 0.4053]. The data were normally distributed; *p*‐values were calculated using one‐way ANOVA with Tukey's post hoc test. (L) Cholesterol concentration in the hippocampus (*n* = 6 mice/group). Brown‐Forsythe test [F(2, 15) = 0.9641, *p* = 0.4038]. The data follow a normal distribution, *p*‐values are calculated using one‐way ANOVA with Tukey's post hoc test. (M) Representative trajectory in the Y‐maze test. (N) Comparison of total moving distances among the three groups (*n* = 13 mice per group). Brown–Forsythe test [F(2, 36) = 2.069, *p* = 0.141]. The data were normally distributed; *p*‐values were calculated using one‐way ANOVA with Tukey's post hoc test. (O) Comparison of the total moving distance in the novel arm among the three groups (*n* = 13 mice/group). Brown–Forsythe test [F(2, 36) = 2.305, *p* = 0.1143]. The data were normally distributed; *p*‐values were calculated using one‐way ANOVA with Tukey's post hoc test. (P) Comparison of time spent in the novel arm among the three groups (*n* = 13 mice per group). Brown–Forsythe test [F(2, 36) = 1.213, *p* = 0.3092]. The data were normally distributed; *p*‐values were calculated using one‐way ANOVA with Tukey's post hoc test. (Q) Representative NORT trajectory. (R) Comparison of total object exploration time among the three groups (*n* = 13 mice per group). Brown–Forsythe test [F(2, 36) = 1.547, *p* = 0.2268]. The data were normally distributed; *p*‐values were calculated using one‐way ANOVA with Tukey's post hoc test. (S) Percentage of time spent exploring a novel object (*n* = 13 mice per group). Brown–Forsythe test [F(2, 36) = 1.965, *p* = 0.1549]. The data were normally distributed; *p*‐values were calculated using one‐way ANOVA with Tukey's post hoc test. (T) Representative trajectory in the OLT. (U) Comparison of the total object exploration time among the three groups (*n* = 13 mice per group). Brown–Forsythe test [F(2, 36) = 0.03795, *p* = 0.9628]. The data were normally distributed; *p*‐values were calculated using one‐way ANOVA with Tukey's post hoc test. (V) Percentage of time spent exploring a novel object (*n* = 13 mice per group). Brown–Forsythe test [F(2, 36) = 2.405, *p* = 0.1046]. The data were normally distributed; *p*‐values were calculated using one‐way ANOVA with Tukey's post hoc test. All data are presented as mean ± SD values. **p < 0.01 *vs* the Saline group; ^#^ *p *< 0.05, ^##^ *p *< 0.01 *vs* the A/S+Saline group.

Hippocampal tissue samples were harvested 12 h after the final administration of minocycline to evaluate its effects. A/S triggered significant neuroinflammation in the hippocampus, as evidenced by elevated levels of pro‐inflammatory cytokines (IL‐6, IL‐1β, and TNF‐α) (Figure 13A–C) and a marked increase in the number of C3^+^/GFAP^+^ cells in the dCA1 (Figure 13D,E). Notably, minocycline treatment effectively reversed these A/S‐induced pathological conditions. Compared with A/S+saline group, there was a significant reduction in the levels of pro‐inflammatory cytokines in the hippocampus of A/S+Minocycline group (IL‐6: *n* = 6 mice/group, one‐way ANOVA, F(2, 15) = 104.2, *p* < 0.01; IL‐1β: *n* = 6 mice/group, one‐way ANOVA, F(2, 15) = 20.85, *p *< 0.01; TNF‐α: *n* = 6 mice/group, one‐way ANOVA, F(2, 15) = 35.6, *p* < 0.01; Figure 13A–C), which was accompanied by a decrease in the density of C3^+^/GFAP^+^ cells in the dCA1 (*n* = 6 mice/group, one‐way ANOVA, F(2, 15) = 52.97, *p *< 0.01; Figure 13D,E).

Importantly, we observed that minocycline treatment reversed the A/S‐induced downregulation of SREBP2 in the hippocampus (*n* = 6 mice/group, one‐way ANOVA, F(2, 15) = 123.1, *p *< 0.01; Figure 13F). As shown in Figure 13G, immunofluorescence staining demonstrated that restoration of SREBP2 expression within the dCA1 astrocyes in the A/S+Minocycline group (density: *n* = 6 mice/group, one‐way ANOVA, F(2, 15) = 74.18, *p* < 0.01, Figure 13H; percentage: *n* = 6 mice/group, one‐way ANOVA, F(2, 15) = 61.22, *p* < 0.01; Figure 13I). Consistent with the restored expression of SREBP2 in the A/S+Minocycline group, neuronal cholesterol levels were also restored, as demonstrated by Filipin staining (*n* = 4 mice/group, one‐way ANOVA, F(2, 9) = 13, *p* < 0.01; Figure 13J,K). ELISA results also confirmed a significant increase in total cholesterol levels in hippocampal tissue (*n* = 6 mice/group, one‐way ANOVA, F(2, 15) = 52.52, Figure 13L).

Finally, we assessed the efficacy of minocycline in reversing surgery‐induced cognitive decline. Representative Y‐maze test trajectories are shown in Figure 13M. No significant differences in total locomotion were observed among the three groups (n = 13 mice per group, one‐way ANOVA, F(2, 36) = 1.261, *p* = 0.2955, Figure 13N). Compared with the A/S+Saline group, mice in the A/S+Minocycline group showed significantly increased distance traveled and time spent in the novel arm (distance: *n* = 13 mice/group, one‐way ANOVA, F(2, 36) = 36.58, *p* < 0.01, Figure 13O; time: *n* = 13 mice/group, one‐way ANOVA, F(2, 36) = 96.55, *p* < 0.01, Figure 13P). In the NORT (Figure 13Q), no significant difference was found in the total time spent exploring objects among the three groups (*n* = 13 mice/group, one‐way ANOVA, F(2, 36) = 0.05861, *p* = 0.9432, Figure 13R), and the time spent exploring the new object was significantly increased in the A/S+Minocycline group compared to the A/S+Saline group (n = 13 mice/group, one‐way ANOVA, F(2, 36) = 149.3, *p* < 0.01, Figure 13S). Similarly, in the OLT (Figure 13T), no significant difference was found in the total time spent exploring objects among the three groups (*n* = 13 mice/group, one‐way ANOVA, F(2, 36) = 0.4565, *p* = 0.6371, Figure 13U). The exploration time for the object in the novel location was significantly longer in the A/S+Minocycline group than in the A/S+Saline group (*n* = 13 mice per group, one‐way ANOVA, F(2, 36) = 250.6, *p* < 0.01; Figure 13V).

Collectively, our results identified a pathogenic cascade from anesthesia‐/surgery‐induced neuroinflammation to SREBP2 downregulation in reactive astrocytes, neuronal cholesterol deficiency, and POCD.

## 3 Discussion

The effects of anesthesia and surgery on neurocognitive outcomes have received extensive attention from anesthesiologists and neuroscientists. To develop unified terms for standardizing cognitive changes after anesthesia and surgery, in 2018, the Nomenclature Consensus Working Group proposed the term PND, which includes preoperative neurocognitive dysfunction, postoperative delirium (POD), delayed neurocognitive recovery (DNR), and POCD [23]. Nevertheless, as we focused on post‐surgery neurocognitive impairment, the term POCD was used in this study. POCD not only hinders the recovery process after surgery but also brings heavy economic and mental burdens onto families. Therefore, exploring the pathogenesis of POCD and developing treatment strategies are particularly important for the perioperative safety of elderly patients.

Decline in learning and memory are important characteristic of aging, with the hippocampus being particularly sensitive to age‐related phenomena [24]. In this study, we verified that hippocampus‐dependent cognitive dysfunction occurred in elderly mice after anesthesia/surgery. Furthermore, anesthesia/surgery inhibited SREBP2 expression in reactive astrocytes in the dCA1 to result in disordered cholesterol synthesis, impaired synaptic plasticity, and inhibition of neuronal excitability. In the intervention experiments, (1) surgery‐induced LTP reduction could be rescued by microinjecting cholesterol into the dCA1 region; (2) overexpression of SREBP2 in dCA1 astrocytes could reverse the disordered cholesterol synthesis, synaptic plasticity impairment, and cognitive dysfunction induced by anesthesia/surgery; and (3) inhibition of reactive astrocytes in the dCA1 could restore the expression of SREBP2, reverse the disordered cholesterol synthesis caused by anesthesia/surgery, and improve synaptic plasticity damage and cognitive dysfunction.

A previous study found that increased neuronal death was not correlated with the degree of aging‐related cognitive impairment, suggesting that other subtle mechanisms also contribute to cognitive impairment in the aging brain [25]. Changes in synaptic connection strength are regarded as foundational for learning and memory [26], while plasma membrane stability is a crucial prerequisite for synaptic transmission. Cholesterol is the main component of the plasma membrane, and in vitro and in vivo evidence suggests that it is an important endogenous factor regulating synaptic transmission and cognitive behavior [27, 28]. Moreover, disrupted cholesterol homeostasis, a key pathological feature of brain aging [14], has been implicated in several neurological disorders, including Alzheimer's disease, Parkinson's disease, and Huntington's disease [29, 30, 31]. Therefore, we wanted to investigate whether cholesterol deficiency constitutes a critical cause of POCD through the disruption of synaptic function in the hippocampus.

The results of the present study revealed that a reduction in cholesterol content in hippocampal neurons was positively correlated with impaired synaptic plasticity and decreased neuronal excitatory transmission caused by anesthesia/surgery. Similarly, the cholesterol deficiency observed in elderly POCD mice was consistent with these phenomena in elderly mice with cognitive decline. LTP data revealed that exogenous cholesterol supplementation into the dCA1 could rapidly rescue the synaptic plasticity impairment caused by anesthesia/surgery. In conclusion, these findings suggest that anesthesia/surgery further exacerbated the cholesterol deficiency in the hippocampus of elderly mice, which might explain why elderly patients are more prone to developing POCD after surgery. Moreover, a reduction in hippocampal cholesterol levels could be a potential predictor of POCD, and targeted supplementation of neuronal cholesterol may emerge as a therapeutic strategy for improving POCD.

Currently, there is no direct method for assessing the cholesterol content in the living human brain. Therefore, minimally invasive methods for detecting the degree of cholesterol metabolism in the brain may be of great significance for the diagnosis of neurological diseases [32]. Cholesterol cannot be directly eliminated from the central nervous system and is mainly decomposed into 24‐OHC by neuronal CYP46A1 [33, 34]. As most of the 24‐OHC in circulation originates from the brain, it has been proposed that the 24‐OHC in plasma or cerebrospinal fluid could serve as a peripheral marker reflecting cholesterol metabolism in the brain [30, 35]. Therefore, the detection of CYP46A1 protein levels and 24‐OHC concentrations has been regarded as a common method for assessing brain cholesterol metabolism. Our findings revealed a significant reduction in 24‐OHC levels in both the hippocampus and peripheral blood of aged mice, with no change in the expression of CYP46A1 protein in hippocampal neurons. Similarly, in the clinical cohort, plasma 24‐OHC levels were significantly decreased in PND patients after surgery. The above evidence demonstrated that anesthesia/surgery did not affect the efficiency of cholesterol metabolism in neurons, and the decrease of 24‐OHC levels in the peripheral circulation was most likely caused by a reduction of cholesterol content in the brain. In conclusion, although we did not test the enzymatic activity of CYP46A1, but combining our experimental evidence and analysis, we believe that the decrease of 24‐OHC content in peripheral plasma can be a potential marker reflecting the risk for POCD mice and PND patients. Of course, testing the enzymatic activity of CYP46A1 is very important; we will explore effective detection methods in future study.

Through evolutionary selection, neurons actively down‐regulate their endogenous cholesterol synthesis capacity to maintain efficient synaptic transmission [36]. The main pathway for maintaining cholesterol homeostasis in neurons is exogenous uptake from astrocytes, rather than endogenous synthesis, resulting in the unique characteristic of cholesterol transfer between astrocytes and neurons. Numerous studies have shown that alterations in the transcriptional characteristics of genes involved in cholesterol synthesis in astrocytes, such as SREBF2, are closely related to disorders of neuronal structure and function [17, 31]. In line with this, our study extends this evidence by showing that anesthesia/surgery specifically downregulates this pathway in hippocampal astrocytes, as evidenced by suppressed SREBF2/SREBP2 expression at both transcriptional (GSEA) and protein levels, with the SREBP2 signal specifically co‐localizing with GFAP but not neurons in the dCA1. Therefore, the observed decrease in neuronal cholesterol in this study cannot be attributed to a reduction in its synthesis, but should be attributed to the disruption of external supply. Critically, astrocyte‐specific knockdown of SREBF2 in the dCA1 of aged mice recapitulated the LTP impairment induced by anesthesia/surgery, and this impairment was largely rescued by exogenous cholesterol. This evidence further confirms that cholesterol deficiency is the primary downstream mechanism through which astrocytic SREBF2 downregulation leads to cognitive impairment. In conclusion, these findings emphasize that cholesterol deficiency in hippocampal neurons, mediated by the downregulation of SREBP2 in astrocytes, is an important cause of neurocognitive dysfunction after anesthesia/surgery.

It is well known that efficient synaptic transmission requires a sensitive response of the postsynaptic membrane to neurotransmitters but relies more critically on presynaptic vesicle neurotransmitter release. The precise fusion of vesicles at the presynaptic membrane determines the release of neurotransmitters and the efficiency of synaptic transmission. Cholesterol is an important component of synaptic vesicles and is crucial for effective synaptic vesicle exocytosis at the presynaptic membrane [37]. Indeed, using transmission electron microscopy, we observed that anesthesia/surgery led to a significant decrease in the density of presynaptic vesicles in the dCA1, which was consistent with the phenomenon of a significant reduction in sEPSC frequency and amplitude of glutamatergic neurons detected using whole‐cell patch clamping. Based on the results of the intervention experiment, our findings indicated that cholesterol deficiency in the hippocampus caused by anesthesia/surgery led to a significant reduction in the quantity of synaptic vesicles and disrupted neurotransmitter function. Our findings are also consistent with the conclusions of Wang et al. [37], which suggests that in‐depth studies on the molecular mechanisms of POCD can be conducted from the novel perspective of cholesterol deficiency, abnormal assembly of the SNARE complex, and abnormal fusion of synaptic vesicles in the future.

Astrocytes are the most abundant type of glial cells in the brain. Recent evidence suggests that neurotoxic reactive astrocytes (A1 phenotype) were more likely to be the effectors of inflammatory microglia and were involved in the pathological process of neurodegenerative diseases and POCD [38, 39]. However, the pathogenicity of A1 phenotype reactive astrocytes arises not only from their active release of neurotoxic substances but is also closely linked to the loss of their essential physiological functions, such as neurotrophic support, synaptogenesis, and neurotransmitter uptake and recycling [40]. Therefore, we propose that in investigating the mechanisms of POCD, the disruption of synaptic homeostasis caused by cholesterol synthesis dysfunction constitutes another critical pathway, apart from neuroinflammation, through which reactive astrocytes contribute to cognitive impairment. This insight emphasized the necessity for future research to adopt a dual perspective—encompassing both “increased toxicity” and “loss of function”—to elucidate the role of A1 phenotype astrocytes in POCD and to identify novel interventional strategies.

Consistent with the key characteristic of insufficient cholesterol synthesis capacity in reactive astrocytes under aging and pathological conditions [41, 42], our findings revealed that morphologically, reactive astrocytes in the dCA1 exhibited more complex processes. Minocycline intervention further demonstrated that surgery‐induced neuroinflammation downregulated SREBP2 in A1 phenotype astrocytes. It is suggested that SREBP2 downregulation may be a conserved and functionally relevant core event in the transformation of astrocytes to harmful phenotypes (such as the A1‐like state) in various pathological conditions. This study extends the pathogenic mechanism of reactive astrocytes from “neurotoxicity” to the dimension of “loss‐of‐function,” and we propose that the consequent disruption of cholesterol metabolism is a key link between neuroinflammation and POCD‐related synaptic deficits. While SREBP2 downregulation may be a feature of reactive astrocytes in other chronic conditions, our data demonstrate that it is a critical and actionable node specifically within the acute pathogenic cascade triggered by anesthesia/surgery in the aged brain. Therefore, targeting the cholesterol metabolic homeostasis of astrocytes may represent a new strategy for shifting the therapeutic paradigm from “anti‐inflammation” to “functional restoration.”

Chemogenetic tools have been reported to be important for regulating astrocyte activity [43, 44]. Based on previous studies [45, 46], the clozapine generated from the reverse metabolism of 2.5 mg/kg CNO is unlikely to account for the observed effects. We found that this intervention attenuated the anesthesia/surgery‐induced upregulation of Complement C3 in astrocytes and the associated neuroinflammation. The current study further elucidates the core mechanism underlying the efficacy of this intervention—inhibiting the activation of astrocytes, especially their A1 phenotype‐associated neurotoxic functions, not only effectively disrupts the pathogenic cascade but also restores key physiological support functions such as cholesterol biosynthesis. This suggests that the benefits of chemogenetic intervention stem not only from the suppression of inflammatory signaling, but more critically, from the restoration of the intrinsic metabolic homeostasis essential for maintaining the tripartite synapse.

Our study had some limitations. (1) The plasma 24‐OHC concentration was tested only on the third day after surgery, and it was insufficient to reveal the dynamic trajectory of its levels. Whether any such changes are temporary fluctuations or a persistent state needs to be clarified in long‐term follow‐up studies with a large sample size. (2) Due to the presence of the BBB, methods for regulating cholesterol metabolism in the brain through peripheral pathways are limited. In this study, LTP in POCD mice improved after stereotaxic injection of cholesterol into the hippocampus; however, this was an invasive procedure unsuitable for clinical application. Therefore, the development of an accurate intracerebral cholesterol transport system for the treatment of POCD will be our main focus in future studies. (3) The APOE genotype is a crucial factor influencing cholesterol metabolism and neurocognitive function. Future clinical studies should incorporate APOE genotyping as a key covariate to provide more conclusive evidence.

**FIGURE 14:**
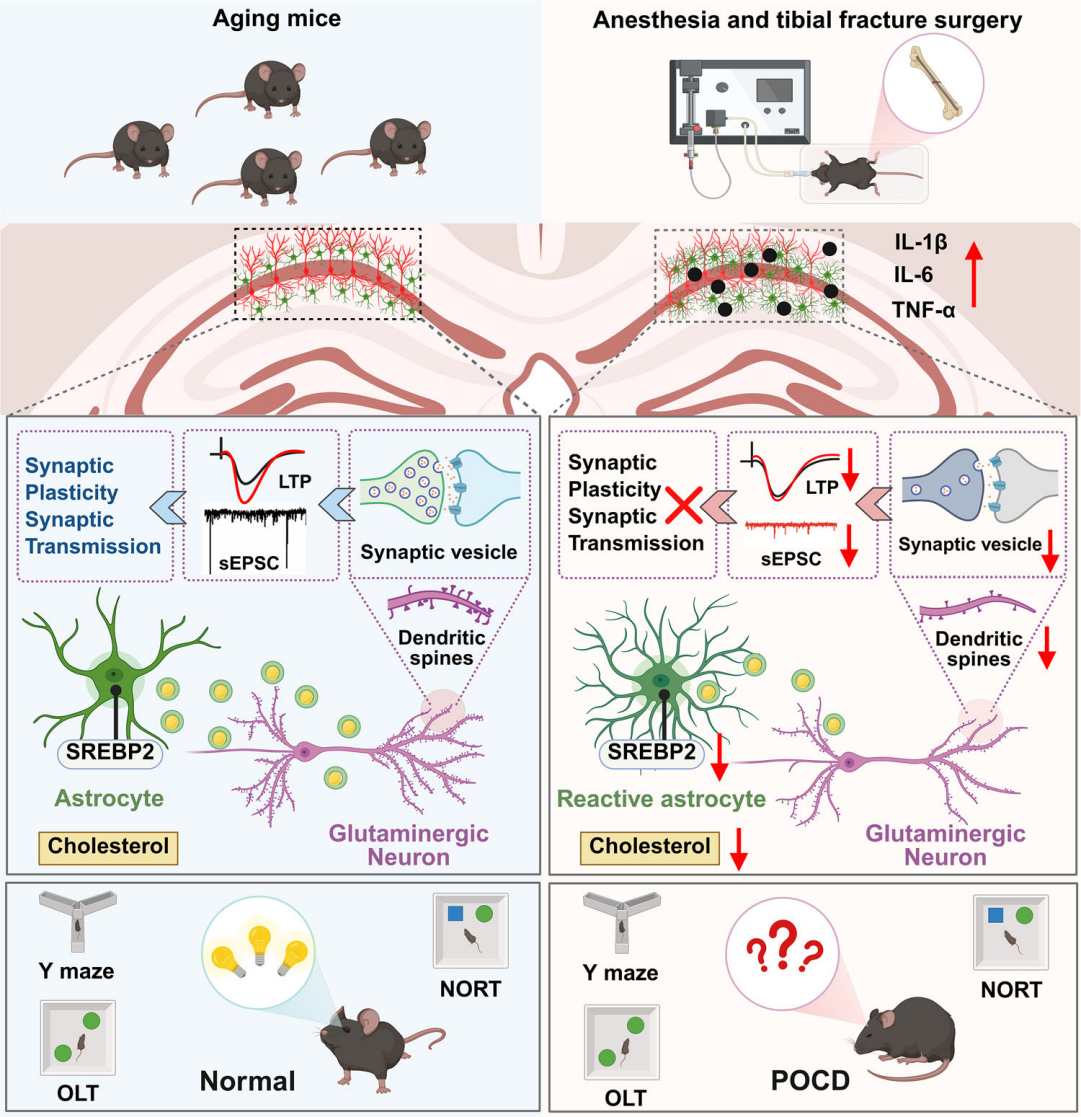
**Graphical Abstract**. The results showed that the reduction of plasma 24 hydroxycholesterol (24‐OHC) was related to the cognitive decline in elderly surgical patients. Neuroinflammation induced by anesthesia and surgery significantly downregulated SREBP2 in hippocampal reactive astrocytes, resulting in reduced cholesterol synthesis. This cholesterol deficiency impaired synaptic plasticity and neurotransmission, thereby driving postoperative cognitive dysfunction (POCD). The present findings suggest that hippocampal cholesterol metabolism is a promising target for the prevention and treatment of POCD, and plasma 24‐OHC may be a potential biomarker for detecting POCD.

In conclusion, this study indicates a critical and novel regulatory node that mediates POCD: anesthesia/surgery causes a deficiency of neuronal cholesterol by inhibiting SREBP2 expression in reactive astrocytes in the hippocampus, thereby leading to impaired synaptic plasticity and inhibition of excitatory synaptic transmission, ultimately resulting in POCD (Figure 14). Our findings provide new insights into the mechanisms underlying POCD from the perspective of brain cholesterol homeostasis and highlight potential preventive and therapeutic strategies.

## 4 Experimental Section

### 4.1 Experimental Animals

Male and female C57BL/6J mice (18‐month‐old) were obtained from the Experimental Animal Centre of Xuzhou Medical University. The GFAP‐Cre mice used in the chemogenetic experiment were obtained from the Jackson Laboratory. All animals had free access to food and water, and were housed at 23°C–25°C temperature and 40%–60% humidity under a 12 h light/dark cycle. All experiments were approved by the Institutional Animal Care and Ethics Committee of Xuzhou Medical University (approval number: 202401T028) and complied with the Guide for the Care and Use of Laboratory Animals of the National Research Council, China.

### 4.2 Construction of the Anesthesia/Surgery Model and Experimental Groups

In experiment 1, the mice were randomly assigned to the control (Ctrl) or anesthesia/surgery (A/S) group. TF surgery under sevoflurane anesthesia was performed as described previously [5]. Briefly, mice received 3.0% sevoflurane for anesthesia induction followed by 1.5% sevoflurane for maintenance. Thereafter, the skin of the left hindlimb was disinfected, a small incision was made to expose the tibia, an intramedullary fixation pin was implanted into the tibial intramedullary cavity, and osteotomy was performed on the middle and distal tibia. Finally, the incision was carefully sutured, and 2% lidocaine was locally applied to reduce postoperative pain. The mice were placed on a heated pad during the operation and returned to their cages for postoperative recovery.

In experiment 2, the mice were divided into A/S+Saline and A/S+Bodipy‐cholesterol groups. Three days after surgery, Bodipy‐cholesterol or saline was injected into the bilateral dorsal hippocampal CA1 (dCA1).

In experiment 3, the mice were divided into four groups: the Ctrl+adeno‐associated virus (AAV) vehicle (VEH) group (Ctrl+VEH); A/S**+**AAV‐VEH group (A/S+VEH); Ctrl+AAV‐SREBF2 group (Ctrl+OE‐SREBF2), and A/S+OE‐SREBF2 group. The AAV‐SREBF2 overexpressing or control AAV vehicle was injected into the bilateral dCA1 21 days before surgery.

In experiment 4, the mice were divided into the AAV‐VEH+Saline, AAV‐shRNA(SREBF2)+Saline (AAV‐shSREBF2+Saline), and AAV‐shSREBF2+Bodipy‐cholesterol groups. Bodipy‐cholesterol or saline was injected into the bilateral dCA1 21 days after AAV injection.

In Experiment 5, the mice were randomly divided into the A/S+hM4D(Gi)/Saline and A/S+hM4D(Gi)/clozapine‐N‐oxide (CNO) (A/S+hM4D(Gi)/CNO) groups. The AAV was injected into the bilateral dCA1 of GFAP‐Cre mice 21 days before surgery. CNO (Med Chem Express, Cat: HY‐17366, Lot: 441162) was dissolved in dimethyl sulfoxide (DMSO) to obtain a stock solution (2.5 mg/ml), which was diluted with normal saline to obtain a working concentration of 0.33 mg/mL. CNO was administered intraperitoneally at a dose of 2.5 mg/kg daily from days 1 to 6 after surgery.

In experiment 6, mice were divided into the Saline, A/S+Saline, and A/S+Minocycline groups. Minocycline (50 mg/kg) was injected intraperitoneally 2 h before surgery and was administered once daily from 1 to 3 days after the operation.

A blinded design was employed to mitigate potential biases. Specifically, the individuals who performed the experiments described above were only responsible for procedures such as anesthesia and surgical modeling, stereotactic injection, intraperitoneal injection, and tissue harvesting, but did not participate in subsequent outcome assessments. All behavioral tests and biochemical, morphological, and electrophysiological analyses were independently conducted by researchers who were blinded to the group assignments. Blinding was not lifted until all data analyses were completed.

### 4.3 Stereotactic Injection Into the Hippocampus

Mice were anesthetized with 3% sevoflurane and fixed in a brain stereotaxator (RWD Life Science Co., Ltd, China) while placed on a heating pad for maintenance of body temperature. The virus was microinjected into the dCA1 for 10 min at 20 nL/min using a 33­gauge syringe needle (Hamilton, 65460‐02, USA) and a pump (Harvard Apparatus, USA). The stereotaxic coordinates relative to Bregma were: anterior‐posterior (AP), ‐1.95 mm; medial‐lateral (ML), ±1.4 mm; dorsal‐ventral (DV), ‐1.55 mm.

The titers of the rAAV2/5‐Gfaabc1D‐SREBF2‐3×Flag‐WPREs (SREBF2 overexpressing viral vector) and rAAV2/5‐Gfaabc1D‐3×Flag‐WPREs (control viral vector) (BrainVTA Biotech, China) were 6.17 × 10^12^ vg/ml and 5.0 × 10^12^ vg/mL, respectively. An Alexa Fluor 488 anti‐DDDDK tagged antibody (Abcam, Cat.: ab245892) was used to bind the Flag sequence. The titers of the rAAV2/5‐Gfaabc1D‐mCherry‐5'miR‐30a‐shRNA(SREBF2)‐3'miR‐30a‐WPREs (SREBF2 inhibitory viral vector) and rAAV2/5‐Gfaabc1D‐mCherry‐5'miR‐30a‐shRNA(scramble)‐3'miR‐30a‐WPREs (control viral vector) (BrainVTA Biotech, China) were 5.83 × 10^12^ vg/mL and 5.16 × 10^12^ vg/mL, respectively. The titer of the rAAV9‐EF1α‐DIO‐hM4D(Gi)‐mCherry chemogenetic viral vector (PackGene Biotech, China) was 1.0 × 10^13^ vg/mL. The injection volume was 200 nL/side in all cases.

Fluorescent Bodipy‐cholesterol (GlpBio Technology, Cat: GC42964) stock solution (500 µg in 1 mL DMSO) was diluted with normal saline to obtain a working concentration of 2 µg/mL for stereotactic brain injection. In experiment 2, we selected two injection points on each side at a distance of over 500 µm (AP: ‐1.95 mm; ML: ±1.1 mm; DV: ‐1.55 mm and AP: ‐1.95 mm; ML: ±1.7 mm; DV: ‐1.55 mm), and injected 100 nL of fluorescent Bodipy‐cholesterol or saline at each injection point. In experiment 4, we performed the bilateral injections of Bodipy‐cholesterol using the same stereotactic coordinates (AP: ‐1.95 mm; ML: ±1.4 mm; DV: ‐1.55 mm). The injection volume was 200 nL/side. The long‐term potentiation (LTP) protocol was commenced 60 min later.

### 4.4 Transcriptome Dataset Collection

Gene Expression Omnibus (GEO) data were obtained using the following search terms: [(POCD) or postoperative cognitive dysfunction or (PND) or perioperative neurocognitive dysfunction]. The mouse hippocampal transcriptome dataset was generated and analyzed using microarray or RNA sequencing (RNA‐seq) platforms. The gene expression profile GSE95426 was retrieved from the GEO database (https://www.ncbi.nlm.nih.gov/geo/query/acc.cgi?acc=GSE95426).

Data extraction was performed using the “GEO query” R package (version 2.70.0), and differential expression analysis was performed using the “limma” package (version 3.58.1). Genes with |log2 fold change|≥2 and *p *< 0.05 were defined as significantly differentially expressed.

Functional enrichment analysis of the differentially expressed genes was performed using the “clusterProfiler” package (v4.10.1) in R. The analysis included Gene Ontology terms covering biological processes, cellular components, and molecular functions, as well as Kyoto Encyclopedia of Genes and Genomes (KEGG) pathway enrichment. Pathways and terms with *p*<0.05 were considered significantly enriched.

Gene Set Enrichment Analysis (GSEA) was used to investigate coordinated expression changes in predefined gene sets between experimental groups. The analysis was performed using the “clusterProfiler” package (v4.10.1) in R, with gene sets obtained from the Molecular Signatures Database (MSigDB), including hallmark, KEGG, and GO collections. Genes were ranked based on their log2 fold change and analyzed using default parameters.

### 4.5 Western Blotting

After 3% sevoflurane anesthesia, hippocampal samples were quickly isolated on ice and homogenized in ice‐cold RIPA lysis buffer (Beyotime) containing phenylmethylsulfonyl fluoride (PMSF). Proteins were separated on 10% gradient sodium dodecyl sulfate‐polyacrylamide electrophoresis (SDS‐PAGE) gels and transferred to PVDF membranes (Merck Millipore, ISEQ00010). The membranes were blocked with 5% skim milk for 2 h and incubated overnight at 4°C with the following primary antibodies: rabbit polyclonal anti‐SREBP2 antibody (ABclonal, Cat.: A25685, 1:1000; HUABIO, Cat.: ER60160, Clone: H650619003, 1:1000), rabbit polyclonal anti‐CYP46A1 antibody (ABclonal, Cat.: A25106; 1:1000), rabbit monoclonal anti‐synaptophysin antibody (ABclonal, Cat.: A19122; Clone: 4000000427; 1:2000), and rabbit monoclonal anti‐postsynaptic density‐95 antibody (HUABIO, Cat.: ET1602‐20; Clone: H681914008; RRID: AB_3069633; 1:2000). β‐actin (mouse monoclonal antibody, HUABIO, Cat.: EM21002; Clone: A2‐F6; RRID: AB_2819164; 1:2000) and β‐tubulin (mouse monoclonal antibody, HUABIO, Cat.: M1305‐2; Clone: A1‐A4; RRID: AB_3073058; 1:2000) were used as internal reference proteins. Membranes were then incubated with the corresponding horseradish peroxidase‐conjugated secondary antibodies (Beyotime, Cat.: A0208/A0216, 1:2000) for 1 h at room temperature. An enhanced chemiluminescence detection system (Beyotime, Cat.: P0018FS) was used to visualize the proteins. Finally, the target protein and internal reference protein bands were quantitatively analyzed using ImageJ 1.4 software (National Institutes of Health, Bethesda, MD, USA).

### 4.6 Immunofluorescence Staining

After 3% sevoflurane anesthesia, the mice were perfused with normal saline through the heart, followed by 4% paraformaldehyde. The brains were fixed in 4% paraformaldehyde for 6–8 h, dehydrated in a 30% sucrose solution for 3 days, and 30 µm coronal sections were then prepared using a freezing microtome (CM1950, Leica, Germany). When a slice containing the entire dCA1 structure began to be exposed, the fifteenth slice was collected. The brain sections were incubated in 10% goat serum for 1 h at 37°C and then at 4°C with the following primary antibodies: rabbit polyclonal anti‐SREBP2 antibody (ABclonal, Cat.: A13049; Clone: 5500038647/5500042291; 1:100); rabbit polyclonal anti‐CYP46A1 antibody (ABclonal, Cat.: A25106; 1:100); mouse monoclonal anti‐GFAP antibody (Cell Signaling Technology, Cat.: 3670; Clone: 9; RRID: AB_561049; 1:200); rabbit polyclonal anti‐Complement C3 antibody (ABclonal, Cat.: A6879, RRID: AB_2767439, 1:100); rabbit monoclonal anti‐Iba‐1 antibody (Abcam, Cat.: ab178846; RRID: AB_2636859; 1:200), rabbit monoclonal anti‐NeuN antibody (Cell Signaling Technology, Cat.: 24307; RRID: 2651140; 1:100) and mouse monoclonal anti‐Olig2 antibody (Cell Signaling Technology, Cat.: 39588; Lot: 1; 1:100). Thereafter, the brain slices were incubated with fluorescent secondary antibodies for 1 h at 37°C. The secondary antibodies were tagged with goat anti‐mouse Alexa 594 (Abcam, Cat.: ab150116, RRID: AB_2650601; 1:500), goat anti‐mouse Alexa 488 (Abcam, Cat.: ab150113, RRID: AB_2576208; 1:500), goat anti‐rabbit Alexa 488 (Abcam, Cat.: ab150077, RRID: AB_2630356; 1:500), goat anti‐rabbit Alexa 594 (Abcam, Cat.: ab150080, RRID: AB_2650602; 1:500) and goat anti‐mouse Alexa 647 (Abcam, Cat.: ab150115, RRID: AB_2687948; 1:500). Finally, DAPI (Abcam, ab104139) was used to stain the nucleus. Images (at 4×, 10×, 20×, and 40× magnification) were obtained using a confocal microscope (FV1000, Olympus).

### 4.7 Assessment of Cholesterol and 24‐Hydroxycholesterol Levels

Hippocampal tissue was rapidly separated, homogenized, and centrifuged. The cholesterol concentration was measured based on the manufacturer's protocols (Solarbio, Cat.: BC1985). Absorbance (optical density, OD) of samples from different groups at 500 nm was measured using a spectrophotometer, and the cholesterol content was calculated by comparing with the cholesterol standard. The total cholesterol concentration was expressed in µmol/g.

The 24‐hydroxycholesterol (24‐OHC) levels in hippocampal tissue and plasma of mice were measured using an ELISA kit (ENZO LIFE SCIENCES, Cat.: ADI‐900‐210‐0001). The experimental procedures are in accordance with the manufacturer's protocol. The absorbance (OD value) was measured at 450 nm, and the sample concentration was calculated.

### 4.8 Quantification of Hippocampal Pro‐Inflammatory Cytokine Levels

Following rapid isolation, hippocampal tissues were immediately homogenized and then centrifuged. Supernatants were collected for analysis, the TNF‐α level was measured using ELISA kit (ABclonal, Cat.: RK00027); the IL‐6 level was measured using ELISA kit (ABclonal, Cat.: RK00008); and the IL‐1β level was measured using ELISA kit (ABclonal, Cat.: RK00006). The absorbance (OD value) at 450 nm was measured using an ELISA plate reader (Multiskan GO, Thermo Scientific, USA).

### 4.9 Transmission Electron Microscopy

Mice were anesthetized using 3% sevoflurane, and hippocampal CA1 tissue cubes (1 × 1 × 1 mm^3^) were quickly prepared on ice and then prefixed for more than 24 h using a mixture of 2.5% glutaraldehyde and 2% paraformaldehyde (1:1). After three washes with 0.1 MPB, the brain tissues were fixed with 1% osmium acid for 2 h. After gradient dehydration with ethanol and acetone, the tissues were immersed and embedded in Epon resin and sliced into 70 nm thick ultrathin sections using an ultrathin slicer [(UC7rt) A‐1170] and stained on copper webs using 4% uranium acetate and lead citrate. A transmission electron microscope (Tecnai G2S Pirit Twin) was used to observe synaptic ultrastructure, and presynaptic vesicle density, postsynaptic density length, and synaptic cleft width were measured using ImageJ 1.4 software (National Institutes of Health, Bethesda, MD, USA). Three mice from each group were used for the experiment. Two cubes from each dCA1 region and two synaptic structures per cube were selected for analysis.

### 4.10 Golgi Apparatus Staining

Mice were anesthetized using 3% sevoflurane, and brain tissue was carefully extracted for processing using a Golgi staining kit (FD NeuroTechnologies, Inc., Cat.: PK401, USA). Briefly, after rinsing with pre‐cooled double steamed water, brains were incubated in an immersion solution consisting of solutions 1 and 2 for 14 days and then in solution 3 for 3 days, according to the manufacturer's instructions. Finally, 120 µm coronal slices were prepared and transferred onto gelatin‐coated slides. After staining, brain slices were dehydrated with alcohol until transparent, and dCA1 region neurons were imaged at 20× and 60× magnification using an Olympus BX53 microscope. Three mice from each group were used for the experiment. Two slices from each mouse and three neurons per brain slice were selected for dendritic spine analysis.

### 4.11 Electrophysiological Assessments

The rAAV‐CaMKIIα‐EGFP‐WPRE AAV (BrainVTA Biotech, China) (viral titer, 5.0 × 10^12^ vg/mL) was injected into the dCA1 region 3 weeks before anesthesia/surgery to label glutamatergic neurons. The protocol for the preparation of brain slices for in vitro electrophysiology has been described previously [5]. A patch electrode (4–8 MΩ) was used for whole‐cell patch clamp recording of labeled hippocampal dCA1 neurons. The sEPSC were recorded at a holding potential of −60 mV. Two criteria had to be met for successful inclusion of recording data: (1) sEPSC amplitude >7 pA; (2) series resistance of recording < 30 MΩ, cells with more than 20% changes in access resistance during an experiment were discarded. The signals were collected using a MultiClamp 700 B amplifier. Data acquisition and analysis were performed using a Digidata 1550B digitizer and pClamp10.7 (Sunnyvale Molecular Devices, USA).

### 4.12 LTP Measurement

Coronal hippocampus tissue sections (300 µm) in frozen section solution pre‐oxygenated with 95% oxygen and 5% carbon dioxide were prepared using a concussion microtome (VT1200S, Leica, Germany). The brain sections were placed in artificial cerebrospinal fluid (ACSF) with continuous oxygenation and incubated at 32°C for 1 h. After incubation, the brain slices were placed in the recording tank for ACSF perfusion, the Schaffer branch was stimulated using bipolar electrodes, and the field excitatory postsynaptic potential (fEPSP) for the CA1 region was recorded using glass microtubules filled with ACSF and recording electrodes. Following a 20 min recording of stable fEPSP (recorded as the stable baseline), three trains of theta burst stimulation (TBS) were administered (frequency, 100 HZ; duration, 1 s; 20 s intervals). Following TBS, fEPSP were recorded for 1 h, and the average fEPSP slope over the last 10 min was analyzed.

### 4.13 Cell‐Based Experiments

#### 4.13.1 Primary Astrocyte Culture and Treatments

(1) The brains of newborn mice were extracted within 24 h under sterile conditions and placed in Petri dishes containing sterile PBS solution. (2) The hippocampal tissue was cut into 0.5 mm^3^ pieces and rinsed with PBS. Precipitates were collected after centrifugation at 1000 rpm for 3 min. (3) The tissues were re‐suspended in buffer containing mixed collagenase and digested by shaking at 37°C for 20 min. (4) DMEM (SH30243.01, Hyclone) containing 10% FBS (S711‐001S, Lonsera) was added to the complete medium to terminate digestion. (5) The suspension was collected and centrifuged at 1000 rpm for 5 min. (6) The obtained cells were inoculated in cell culture dishes and cultured in an incubator with 5% CO_2_ volume fraction at 37°C and saturated humidity. (7) The culture medium was changed once every 3 days. (8) When the cells reached 90% confluence, they were fixed using 4% paraformaldehyde for 15 min, then incubated with an anti‐GFAP antibody (Cell Signaling Technology, Cat.: 3670; 1:400) at 4°C overnight for astrocyte identification, and then observed using an inverted phase‐contrast microscope. Control group: no treatment; lipopolysaccharide (LPS) group: astrocytes were treated with 10 ng/mL LPS for 12 h.

#### 4.13.2 SREBP2 Expression in Cultured Astrocytes

First, the supernatant from each well was discarded, and the cells were washed with PBS. Trypsin (0.25%) was then added to digest the cells. Once cell rounding was observable, medium containing FBS was added to terminate the digestion. The samples were centrifuged at 1200 rpm for 3 min to collect the cell precipitates, and immunoblotting was performed as previously described using primary anti‐SREBP2 rabbit polyclonal (Origene, Cat.:TA381992; 1:1000) and anti‐GAPDH (Bioss, Cat.: bsm‐33033 m; 1:10000) antibodies.

#### 4.13.3 Cholesterol Detection in Cultured Astrocytes and Culture Medium

Extract solution (mL) at a ratio of 500:1 of the cell quantity (10^4^ cells) was added, and the cells were lysed by ultrasonication (300 W; ultrasonication for 2 s at 3 s intervals; total time, 3 min). The samples were then centrifuged at 10 000 rpm for 10 min at 4°C, and the supernatant was maintained on ice until further analysis. The cholesterol in the medium was directly extracted for determination, and the cholesterol content of the samples was calculated according to the above protocol (Solarbio, Cat.: BC1985, China).

### 4.14 Filipin III Staining

Filipin III is a fluorescent probe widely used for assessing the localization of cholesterol on membranes. For neuronal cholesterol staining, hippocampal slices were labeled overnight with 50 µg/mL Filipin III (APE×BIO, B6034) in a dark environment at 4°C. Images (magnification, 60×) of Filipin III‐positive neurons in the dCA1 region were captured using a confocal microscope (FV1000, Olympus).

### 4.15 Behavioral Tests

The open field test (OFT) was used to assess whether locomotor ability was affected by TF surgery. Mice were initially placed in a 50 × 50 × 50 cm^3^ box for 10 min of acclimatization and allowed to freely explore the open field. The total distance moved was recorded using the ANY‐maze video tracking software (ANY‐maze, Stoelting Co., IL, USA). At the end of each exploration, the field was wiped with 75% ethanol to eliminate any odors that could affect subsequent tests.

The Y‐maze consists of three arms, and the test is divided into training and testing phases. During the training phase, one chosen arm of the Y‐maze was closed, leaving the starting arm and the other arm for exploration. Each mouse was placed at the same position of the starting arm and allowed to explore freely for 10 min. The test phase was conducted 2 h later. The new arm was opened, and mice were placed in the start arm and allowed to explore all three arms freely for 5 min. The distance and time of movement in the new arm were recorded using the ANY‐maze video tracking software (ANY‐maze, Stoelting Co., IL, USA). At the end of each exploration, all arms were wiped with 70% alcohol.

For the novel object recognition test (NORT), mice were free to explore the open field for 10 min in the training phase, and the time taken to explore two objects (ob1 and ob2) was recorded. Two hours later, mice were returned to the open field with one of the objects (ob2) replaced by a new object (ob3), and the time spent exploring ob1 and ob3 was recorded. The percentage of exploration time was defined as the exploration time for one object/total exploration time for both objects × 100%, where the percentage of exploration of ob3 indicates the preference for a new object. At the end of each exploration, the experimental sites and objects were cleaned with 75% ethanol.

For the object location test (OLT), mice were exposed to two objects (ob1 and ob2) in an open field in the training phase, and the time spent exploring each object was recorded. Two hours later, one object (ob2) was moved to a new location, and the time spent exploring the unmoved object (ob1) and the moved object (moved ob2) was recorded. The exploration time percentage was defined as the exploration time for one object/total exploration time for both objects × 100%, where the exploration percentage for the moving object (moved ob2) indicates the preference for a new location. At the end of each exploration, the experimental sites and objects were cleaned with 75% ethanol.

### 4.16 Clinical Data Collection

This study included patients who underwent lower limb orthopedic surgery (total knee arthroplasty, total hip arthroplasty, femoral neck fracture, or tibiofibular fracture surgery) under general anesthesia at the First Affiliated Hospital of Nanjing Medical University between August 1, 2025, and September 30, 2025. The study protocol was approved by the Ethics Committee of the Medical Institution of the First Affiliated Hospital of Nanjing Medical University (2025‐SR‐430).

The inclusion criteria were age 65–80 years and American Society of Anesthesiologists (ASA) grade I–III. All patients were able to cooperate during the neuropsychological tests. We excluded patients meeting any of the following criteria: (1) Mini‐Mental State Evaluation (MMSE) score ≤ 24 points (educational duration > 6 years); (2) abnormal cholesterol levels before surgery; (3) diagnosed with severe heart, lung, liver, or kidney disease; (4) history of neurological or mental disorders; (5) history of trauma or emergency surgery; (6) unwillingness to participate in the study.

Anesthesia was induced using 0.3 mg/kg remimazolam, 0.3 µg/kg sufentanil, and 0.6 mg/kg rocuronium. The respiratory parameters for mechanical ventilation were as follows: inspired oxygen concentration, 60%; tidal volume, 6–8 mL/kg; inspiratory/expiratory ratio, 1:2; positive end‐expiratory pressure, 5 cm H_2_O; and respiratory rate set to 10–15 times/min. The end‐tidal carbon dioxide partial pressure was maintained at 35–45 mmHg. Anesthesia was maintained using 1.0%–1.5% sevoflurane or 2.0%–3.0% desflurane; 2–6 mg/(kg·h) propofol, 0.05–0.25 µg/(kg·min) remifentanil, 5–10 µg/(kg·min) rocuronium, and 0.2–0.7 µg/(kg·h) dexmedetomidine. The bispectral index was maintained at 40–60 during surgery. Blood pressure and heart rate were maintained within −10% to 20% of the baseline level.

The MMSE was used to assess cognitive function one day before the surgery and on the third day after the surgery. Based on the postoperative MMSE scores [47], patients were divided into the PND and NPND groups. Peripheral blood (5 mL) was collected from patients before and 3 days after surgery. Plasma samples were obtained using centrifugation, and an ELISA kit (ENZO LIFE SCIENCES, Cat.: ADI‐900‐210‐0001) was used to measure plasma 24‐OHC levels.

### 4.17 Statistical Analysis

Data were analyzed using GraphPad Prism 8.0 (GraphPad Software Inc., USA). Error bars in the figure legends represent standard deviations (SDs), and n represents the number of samples or independent experiments. Western blotting and some immunofluorescence data were normalized.

For comparisons between two groups, the unpaired t‐test was used for normally distributed data with equal variances, Welch's *t*‐test was used for normally distributed data with unequal variances, and the Mann–Whitney *U* test was used for non‐normally distributed data. Variance homogeneity was assessed using F‐tests.

For multiple group comparisons, one‐way analysis of variance (ANOVA) followed by Tukey's post hoc test was used for normally distributed data, and the Kruskal–Wallis test followed by Dunn's post hoc test was applied to non‐normally distributed data. Equal variances were confirmed using the Brown–Forsythe test.

Sholl analysis data were compared using two‐way ANOVA with Bonferroni's post hoc test. If the non‐normally distributed data passed the normality test after undergoing logarithmic transformation (Y = Log(Y + 1)), two‐way ANOVA was performed on the transformed dataset.

In the statistical analysis of clinical data, the chi‐square test was used for normally distributed clinical count data. Two‐tailed tests and *α* = 0.05 were used for all statistical analyses. Normally distributed data are presented as mean ± SD; data that were not normally distributed are presented as median and interquartile range (IQR). Statistical significance was set at *p *< 0.05.

## Conflicts of Interest

The authors declare no conflict of interest.

## Data Availability

The data that support the conclusions of this manuscript will be made available upon reasonable request.
